# Muscle Proteome Analysis of Facioscapulohumeral Dystrophy Patients Reveals a Metabolic Rewiring Promoting Oxidative/Reductive Stress Contributing to the Loss of Muscle Function

**DOI:** 10.3390/antiox13111406

**Published:** 2024-11-16

**Authors:** Manuela Moriggi, Lucia Ruggiero, Enrica Torretta, Dario Zoppi, Beatrice Arosio, Evelyn Ferri, Alessandra Castegna, Chiara Fiorillo, Cecilia Gelfi, Daniele Capitanio

**Affiliations:** 1 Department of Biomedical Sciences for Health, University of Milan, Via Luigi Mangiagalli 31, 20133 Milan, Italy; manuela.moriggi@unimi.it (M.M.); daniele.capitanio@unimi.it (D.C.); 2 Department of Neurosciences, Reproductive and Odontostomatological Sciences, University of Naples “Federico II”, Via Sergio Pansini 5, 80131 Naples, Italy; lucia.ruggiero@unina.it (L.R.); d.zoppi@studenti.unina.it (D.Z.); 3 Laboratory of Proteomics and Lipidomics, IRCCS Orthopedic Institute Galeazzi, Via R. Galeazzi 4, 20161 Milan, Italy; enrica.torretta@grupposandonato.it; 4 Department of Clinical Sciences and Community Health, University of Milan, Via della Commenda 19, 20122 Milan, Italy; beatrice.arosio@unimi.it; 5 IRCCS Ca’ Granda Ospedale Maggiore Policlinico Foundation, Via Francesco Sforza 35, 20122 Milan, Italy; evelyn.ferri@policlinico.mi.it; 6 Department of Biosciences, Biotechnologies and Environment, University of Bari ALDO MORO, Via Orabona 4, 70125 Bari, Italy; alessandra.castegna@uniba.it; 7 Child Neuropsychiatric Unit, IRCCS Istituto Giannina Gaslini, DINOGMI-University of Genova, Via Gerolamo Gaslini 5, 16147 Genova, Italy; chiara.fiorillo@edu.unige.it

**Keywords:** facioscapulohumeral muscular dystrophy, hexosamine biosynthetic pathway, metabolic rewiring, proteomics, redox cofactors

## Abstract

Facioscapulohumeral muscular dystrophy (FSHD) is caused by the epigenetic de-repression of the double homeobox 4 (DUX4) gene, leading to asymmetric muscle weakness and atrophy that begins in the facial and scapular muscles and progresses to the lower limbs. This incurable condition can severely impair muscle function, ultimately resulting in a loss of ambulation. A thorough analysis of molecular factors associated with the varying degrees of muscle impairment in FSHD is still lacking. This study investigates the molecular mechanisms and biomarkers in the biceps brachii of FSHD patients, classified according to the FSHD clinical score, the A-B-C-D classification scheme, and global proteomic variation. Our findings reveal distinct metabolic signatures and compensatory responses in patients. In severe cases, we observe pronounced metabolic dysfunction, marked by dysregulated glycolysis, activation of the reductive pentose phosphate pathway (PPP), a shift toward a reductive TCA cycle, suppression of oxidative phosphorylation, and an overproduction of antioxidants that is not matched by an increase in the redox cofactors needed for their function. This imbalance culminates in reductive stress, exacerbating muscle wasting and inflammation. In contrast, mild cases show metabolic adaptations that mitigate stress by activating polyols and the oxidative PPP, preserving partial energy flow through the oxidative TCA cycle, which supports mitochondrial function and energy balance. Furthermore, activation of the hexosamine biosynthetic pathway promotes autophagy, protecting muscle cells from apoptosis. In conclusion, our proteomic data indicate that specific metabolic alterations characterize both mild and severe FSHD patients. Molecules identified in mild cases may represent potential diagnostic and therapeutic targets for FSHD.

## 1. Introduction

Facioscapulohumeral muscular dystrophy (FSHD) is a progressive disease exhibiting significant variability both within and between families, with manifestations ranging from asymptomatic to wheelchair dependence. The most common form (FSHD1) is characterized by symmetrical or asymmetrical muscle loss and weakness caused by a reduction in D4Z4 macrosatellite repeats (from 1 to 10 units) which results in epigenetic de-repression at chromosome 4q35 resulting in the aberrant activation of specific genes regulated by the transcription factor double homeobox 4 (DUX4) [1,2,3,4,5,6,7]. The role of DUX4 is still under investigation, with hypotheses suggesting direct interference with embryonic/fetal development or growth processes, impairing satellite cell function [8,9,10], or indirect contribution to myofiber damage and satellite cell dysfunction in the presence of pathogenic mutations in DUX4-related genes [11,12,13]. FSHD pathology encompasses diminished myogenesis, increased ROS production, and inflammation, evidenced by network analyses of pluripotent stem cells from FSHD patients revealing dysregulation in genes crucial for sarcomere structure, muscle contraction, and extracellular matrix organization [14].

Furthermore, aberrant expression of other genes at the 4q35 locus, such as FSHD region gene 1 and 2 (FRG1, FRG2), solute carrier family 25 member 4 (SLC25A4, ANT1), and FAT atypical cadherin 1 (FAT1), has been identified as a set of disease modifiers. These genes influence disease onset, muscle selection, and severity by modulating histone-lysine N-methyltransferase activity, mitochondrial function, and cell-to-cell interactions [15,16,17,18,19]. DUX4 prompts p53-dependent apoptosis, mitochondrial dysfunction, and oxidative stress, exacerbating muscle damage through disruption of the glutathione redox pathway and induction of hypoxia-inducible factor 1 subunit alpha signaling, suggesting the potential roles of methyltransferase activity, mitochondrial import, and cell-to-cell contact in this disease [20,21,22,23,24,25,26,27,28].

Proteomic studies on biological fluids from FSHD patients have identified dysregulated circulating miRNAs and proteins, including S100A8, creatine kinase MM and MB isoforms, carbonic anhydrase III, troponin I type 2, tissue-type plasminogen activator, myoglobin, epidermal growth factor, chemokine (C-C motif) ligand 2, CD40 ligand, and vitronectin [29,30,31,32]. Muscle tissue and cell model studies have further revealed disruptions in mRNA processing, stress response pathways, the ubiquitin/proteasome system, as well as the disturbance of several caveolar proteins and myoblast dynamics [19,33,34,35,36,37] contributing to highlight the morphological and structural changes typical of FSHD. However, while invaluable for understanding pathomechanisms, cell models have limitations in replicating the complexity of human muscle tissue and may underestimate the impact of the surrounding environment in which muscle fibers are embedded. Discrepancies between gene expression and protein levels in FSHD muscle underscore the necessity for novel diagnostic modalities [19,33,36]. Recent advancements, including longitudinal RNA expression profiling and comparative proteomic analyses of muscle extracts and interstitial fluids, have shed light on disease activity and potential therapeutic targets [14,29,34,38,39].

The present study focuses on mechanisms and molecular markers in the *biceps brachii* muscle of FSHD patients, analyzed based on their severity score using a combination of proteome, bioinformatic analysis and gene expression of target genes, unraveling novel mechanisms involved in FSHD. Metabolic dysregulation, associated with DUX4 activity, varies between mild and severe FSHD patients, with mild cases exhibiting compensatory mechanisms to mitigate metabolic stress, inflammation, and oxidative damage. In contrast, severe patients demonstrate a severe failure of central cell metabolism, marked by dysregulated glycolysis, activation of the reductive pentose phosphate pathway (PPP), TCA cycle rewiring, oxidative phosphorylation depression, hexosamine biosynthetic pathway (HBP) inhibition, and hyaluronic acid overproduction and intermediates imbalance exacerbating muscle wasting and inflammation. Comparative analyses reveal distinct metabolic signatures and compensatory responses between mild and severe cases, providing insights into potential therapeutic interventions targeting metabolic dysfunction and inflammation.

## 2. Materials and Methods

The disclosure of this study was approved by patients via informed consent. The study was approved by the Ethical Committee at the Federico II University, protocol number 20/2013.

### 2.1. Study Design and Muscle Biopsies

Genetic, clinical, and histopathological data of 14 patients affected by FSHD were collected from 2013 to 2014. Patients were re-evaluated and classified based on the FSHD score [40] and according to the A-B-C-D scheme [41].

The patients included 5 females and 4 males, aged between 16 and 64 years (average age 43 years), with an age at onset of the disease from 7 to 50 years and a disease score from a minimum of 4 to a maximum of 7 points of severity (average score 5.5). The size of the EcoR1 fragment ranged from a minimum of 17 to a maximum of 35 kb. Among these, 4 patients were classified as A2 (mild classic FSHD), with a severity score between 4 and 7 and an EcoR1 fragment between 30 and 35 kb. Finally, 1 patient was classified as D1 (atypical FSHD), as the fragment was only 35 kb and the disease score was 6. The other 5 patients (male/females) were classified as A (severe classic FSHD), with a severity score ranging from 8 to 15 and the EcoR1 fragment from 17 to 32 kb. A summary of the clinical characteristics is schematically represented in Supplementary Table S1.

Each patient underwent a muscle biopsy from the *biceps brachii* muscle of the most-affected side. The muscle biopsies were analyzed to highlight the histomorphological and histochemical characteristics with the following staining according to standard procedure: hematoxylin–eosin; modified Gomori trichrome, myofibrillar ATPase pH 9.4–4.6–4.3; acid phosphatase; succinic dehydrogenase—SDH; cytochrome C oxidase—COX; NADH—tetrazolium-reductase; periodic acid shift—PAS; oil red O—ORO; phosphorylase; non-specific esterase. The typical histopathological alterations of muscular dystrophies were highlighted in the majority, such as the variability of the fiber caliber and the presence of necrotic and degenerative phenomena (9 out of 9 cases). Inflammatory cellular infiltrates (6/9) and nuclear centralizations (6/9) were common. Furthermore, signs of muscle damage that were not exclusively primitive were noted, such as the presence of small, angulated fibers indicative of neurogenic damage in 8 cases. Finally, 5 biopsies showed a reduction in oxidative reactivity assessed with cytochrome oxidase (COX) and in 2 biopsies vacuoles and/or lipid accumulation were observed. Severe patients revealed typical histopathological alterations of muscular dystrophies such as internal nuclei, fiber size variability, necrosis, and degeneration with a concomitant increase in connective tissue. The results are shown in Supplementary Figure S1 and Table S1.

In addition, muscle biopsies were performed from the *biceps brachii* muscle of 6 control subjects aged between 38 and 55 years (CTR). Among these, 4 patients were characterized by hyperCKemia in the absence of muscular symptoms, while 2 cases were healthy relatives of patients not included in the study. All biopsies from control subjects were normal or negative for any specific muscle pathology.

### 2.2. Protein Extraction for 2D-DIGE and Label-Free LC–ESI–MS/MS Analysis

For label-free proteomics analysis, an aliquot of each frozen muscle was suspended in 2% SDS, 100 mM Tris-HCl pH 7.6, 0.1 M dithiothreitol (DTT), and 1 mM phenylmethanesulfonyl fluoride (PMSF) and sonicated on ice until completely dissolved. Lysates were clarified by centrifugation at 16,000× *g* for 5 min at 20 °C.

For 2D-DIGE, after tissue homogenization, each sample from each subject was suspended in lysis buffer (7M urea, 2M thiourea, 4%3-[(3-cholamidopropyl) dimethylammonio]-1-propanesulfonate (CHAPS), 30 mM Tris, and 1 mM PMSF) and solubilized by sonication on ice. Proteins were selectively precipitated using the PlusOne 2D-Clean up Kit (GE Healthcare, Little Chalfont, UK), in order to remove non-protein impurities, and resuspended in lysis buffer. The pH of the protein extracts was adjusted to pH 8.5 by the addition of 1 M NaOH.

The protein concentration for both 2D-DIGE and label-free samples was determined using the PlusOne 2D-Quant Kit (GE Healthcare, Little Chalfont, UK).

### 2.3. Two-Dimensional Difference in Gel Electrophoresis

Soluble extracts from each frozen muscle were analyzed by quantitative 2D-DIGE followed by mass spectrometry. Protein minimal labeling with cyanine dyes (Cy3 and Cy5), 2D separation, and analyses were performed, as described previously [42]. Representative 2D maps are shown in Supplementary Figure S2. Each individual sample was run in duplicate to minimize the inter-gel variability and increase the results’ reliability. Statistically significant differences were calculated using the ANOVA test followed by the Tukey post-hoc test with a *p*-value threshold of 0,05. False discovery rate (FDR) analysis was applied to reduce the overall error. The principal component analysis was performed on the dataset of significantly altered proteins using the EDA (Extended Data Analysis) module included in the DeCyder 2D image analysis software (GE Healthcare, version 7.0).

Proteins of interest were identified by matrix-assisted laser desorption/ionization–time-of-flight (MALDI-ToF)/MS, as previously described [43].

### 2.4. Label-Free Liquid Chromatography with Tandem Mass Spectrometry

Protein extracts (200 µg for each sample) were processed following the filter-aided sample preparation (FASP) protocol [44]. Peptide samples were concentrated, separated, analyzed, and identified as previously described [45]. Statistical analysis was performed using an ANOVA test followed by a Tukey post-hoc test with a *p*-value threshold of 0.05, and the results showed the variation in protein expression between mild FSHD vs. CTR and severe FSHD vs. CTR. False positives were excluded using the Benjamini–Hochberg false discovery rate test.

### 2.5. Ingenuity Pathway Analysis

Functional and network analyses of statistically significant protein expression changes were performed through Ingenuity Pathway Analysis (IPA) software (Qiagen, Hilden, Germany, Summer Release 2024). In brief, datasets with protein identifiers, statistical test *p*-values, and fold-change values calculated using label-free LC-ESI-MS/MS were analyzed by IPA. The “core analysis” function was used for data interpretation through the analysis of biological processes, canonical pathways, diseases, and bio functions enriched with differentially regulated proteins. Then the “comparison analysis” function was used to visualize and identify significant proteins or regulators across experimental conditions. *p*-values were calculated using a right-tailed Fisher’s exact test. The activation z-score was used to predict the activation/inhibition of a pathway/function/disease and bio functions [46]. A Fisher’s exact test *p*-value < 0.05 and a z-score ≤−2 and ≥2 were considered statistically significant.

### 2.6. Immunoblotting

Protein extracts (50 µg) from CTR and mild and severe FSHD muscle samples were loaded and resolved on 10–16% and 12–18% gradient polyacrylamide gels. Blots were incubated with antibodies from Santa Cruz Biotechnology (sc, Dallas, TX, USA), Cell Signaling Technology (cs, Danvers, MA, USA), Sigma-Aldrich (St. Louis, MO, USA), and Invitrogen: mouse monoclonal anti-Paired Box 7 (PAX7, sc-81648, 1:500), rabbit polyclonal anti-myogenin (sc-576, 1:500), rabbit polyclonal anti-glutamine synthetase (GLUL, sc-6640R, 1:500), mouse monoclonal anti-O-GlcNac (cs-9875, 1:1000), rabbit monoclonal anti-glutamine:fructose-6-phosphate aminotransferase 1 (GFAT1, cs-5322, 1:1000), rabbit monoclonal anti-O-GlcNAc transferase (OGT, cs-24083, 1:1000), rabbit anti-O-GlcNAcase (OGA, Sigma-Aldrich, SAB4200267, 1:1000), rabbit anti-hyaluronan synthase 1 (HAS1, Sigma-Aldrich, SAB4300848, 1:1000), rabbit polyclonal anti-STT3B (Invitrogen PA5-106380, 1:1000), rabbit monoclonal anti-lysosome-associated membrane protein 2 (LAMP2, cs-49067, 1:1000), rabbit polyclonal anti-caspase-3 (cs-9662, 1:1000), rabbit polyclonal anti-LC3B (cs-2775, 1:1000), rabbit monoclonal anti-HSC70 (cs-8444, 1:1000), and rabbit polyclonal anti-p53 (sc-6243, 1:500). After washing, membranes were incubated with an anti-rabbit (GE Healthcare, 1:10,000) or anti-mouse (Jackson ImmunoResearch, Ely, UK, 1:5000) secondary antibody conjugated with horseradish peroxidase. Signals were visualized by chemiluminescence using the ECL Prime detection kit and the Image Quant LAS 4000 (GE Healthcare) analysis system. Band quantification was performed using the Image Quant TL v. 8.1 (GE Healthcare) software followed by statistical analysis (Student’s *t*-test, n = 2, *p*-value < 0.05). Band intensities were normalized against the total amount of proteins stained by the Sypro ruby total-protein stain.

### 2.7. Intracellular NAD^+^ and NADH Quantification

Intracellular NAD^+^ and NADH were measured with the NAD^+^/NADH Quantification Colorimetric Kit (Sigma-Aldrich, MAK037) according to the manufacturer’s instructions. Briefly, pooled tissues from controls (CTR) and mild and severe FSHD patients were prepared using an NAD^+^/NADH extraction solution. The supernatant was retained after homogenization and centrifugation. To measure the total NAD^+^/NADH, 50 μL of the tissue suspension was added to a 96-well plate, in triplicate for each sample. For NADH measurement, the tissue suspension was incubated at 60 °C for 30 min, and 50 μL was added to a 96-well plate. Subsequently, 100 μL of master reaction mix was added to the samples, which were then incubated at room temperature for 5 min. Finally, 10 μL of NADH developer was added to each well, and the mixture was incubated at room temperature for 1–4 h. A standard curve was generated and measured concurrently with the samples. Absorbance values were measured at 450 nm using a plate reader. The NAD^+^/NADH ratio was calculated using the standard curves for NAD^+^ and NADH.

### 2.8. Intracellular NADP^+^ and NADPH Quantification

Intracellular NADP^+^ and NADPH levels were measured using the NADP^+^/NADPH Assay Kit (Sigma-Aldrich, MAK479) according to the manufacturer’s instructions. Determination of both NADP^+^ and NADPH concentrations required extractions from two separate samples. Briefly, pooled tissues from the control (CTR), mild FSHD, and severe FSHD groups were prepared using NADP^+^ and NADPH extraction buffers. The prepared tissue suspension was incubated at 60 °C for 5 min. To neutralize the extracts, 20 μL of assay buffer and 100 μL of the opposite extraction buffer were added. After centrifugation, 40 μL of the prepared tissue suspension was added to a 96-well plate, in triplicate for each sample, to measure NADP^+^ and NADPH.

Subsequently, 80 μL of working reagent was added, and the optical density was immediately measured at 565 nm for the time “zero” (OD0). The plate was incubated for 30 min at room temperature, and after incubation, the absorbance was measured again at 565 nm (OD30). A standard curve was generated and measured concurrently with the samples. The NADP^+^/NADPH ratio was calculated using the standard curves for NADP^+^ and NADPH.

### 2.9. Gene Expression

Total RNA was extracted from 6 to 18 mg of muscle biopsies using TRItidy G™ (PanReac AppliChem, ITW reagents, Italy), according to the manufacturer’s instructions. The RNA concentration was determined using a NanoPhotometer N60 spectrophotometer (Implen GmbH, München, Germany). From one to two micrograms of total RNA were reverse-transcribed using the SuperScript VILOTM cDNA Synthesis Kit (Invitrogen by Thermo Fisher Scientific, Waltham, MA, USA).

Quantitative PCR analysis was performed in the OpenArray^®^ QuantStudio 12K Flex Real-Time PCR System (Applied Biosystems by Thermo Fisher Scientific, Waltham, MA, USA), using the commercial probes shown in Supplementary Table S2. The GAPDH, ACTB, and 18S genes were included in the OpenArray^®^ chip and used as housekeeping endogenous control genes. A total of 1.2 µL corresponding to 60–120 ng of each cDNA sample was added to 1.3 µL of PCR-grade water and 2.5 µL of TaqManTM OpenArray^®^ Real-Time PCR Master Mix (Applied Biosystems by Thermo Fisher Scientific, Waltham, MA, USA). Each analysis was conducted in duplicate.

Statistically significant differences were computed by analysis of variance (ANOVA) and Bonferroni tests (*p* < 0.05) for the comparison between the following: CTR vs. FSHD mild, CTR vs. FSHD severe. In cases where the ANOVA test was not applicable, the non-parametric Kruskal–Wallis test was used. False discovery rate (FDR) analysis was applied to correct for multiple tests to reduce the overall error.

## 3. Results

The present study reports on protein alterations observed in *biceps brachii* muscle extracts from nine FSHD patients exhibiting a mild form of the disease, characterized by a FSHD score between 4 and 7, and five patients with severe disease, with a disease score ranging from 8 to 15, compared to six age-matched healthy controls.

### 3.1. Sample Classification Based on Proteome Analysis by 2D-DIGE

**Figure 1:**
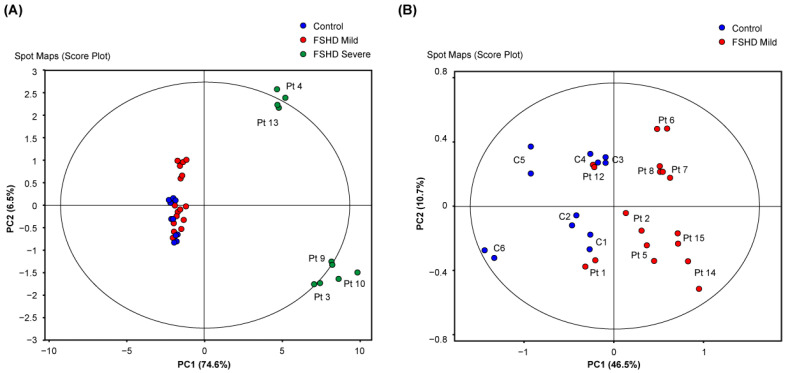
Principal component analysis (PCA) score plots showing the spatial distribution of (**A**) control (blue circles, n = 6), mild (red circles, n = 9), and severe FSHD patients (green circles, n = 5) and (**B**) control and mild FSHD patients only, according to the 2D-DIGE proteomic profile of the *biceps brachii* muscle. The amount of variance explained by each component is indicated on the PC1 and PC2 axes. Each sample was analyzed in duplicate.

The principal component analysis conducted on the altered proteins in the 2D-DIGE analysis revealed a marked difference in protein expression between patients with the highest FSHD scores and all other samples (Figure 1A). After excluding the severe patients from the analysis, further differences emerged between mild FSHD patients and controls (Figure 1B), with only partial overlap of two patients (Pt 1 and 12) with the control group.

### 3.2. Proteome Analysis and Molecular Fingerprint of Known DUX4 Targets

For proteome analysis, 2D-DIGE and liquid chromatography coupled with electrospray tandem mass spectrometry (LC-ESI-MS/MS) were adopted. Among the 1732 proteins identified through 2D-DIGE and LC–ESI–MS/MS, 228 proteins in mild and 668 proteins in severe cases were altered compared to CTR (ANOVA test, *p*-value < 0.05). The identification data for the altered proteins detected by 2D-DIGE and LC–ESI–MS/MS are presented in Supplementary Files as registered in the UNIMI dataverse repository (https://doi.org/10.13130/RD_UNIMI/KTS29V, accessed on 14 November 2024).

**Figure 2:**
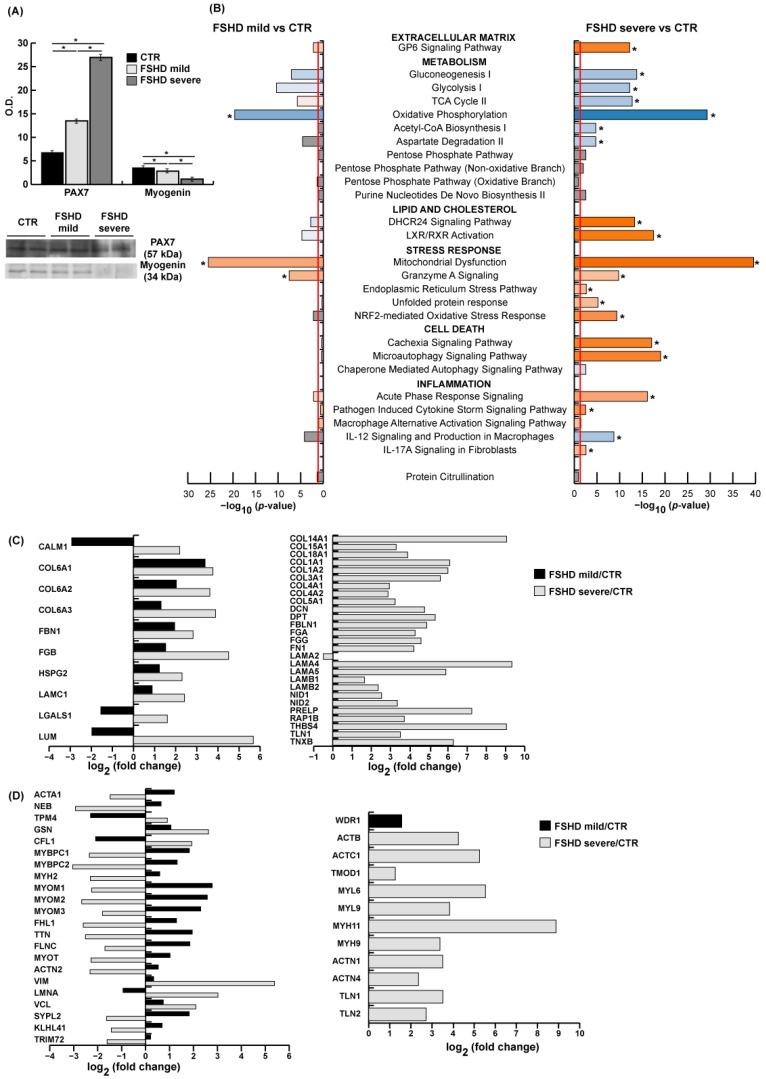
(**A**) Representative bar graph (means ± SD) and immunoblot images of PAX7 and myogenin from healthy controls (CTR, black bars) and mild and severe FSHD patients (gray bars) (n = 2; mean ± SD; Student’s *t*-test, *p* < 0.05). Data were normalized against the total amount of loaded proteins stained with Sypro Ruby. O.D. = optical density. * = statistically significant. Full-length images are available in Supplementary Figure S5. (**B**) IPA analysis showing the over-represented canonical pathways in mild and severe FSHD patients compared to controls ordered by *p*-value and z-score. The significance threshold, indicated by an orange vertical line, is set at *p* = 0.05. The color orange indicates predicted pathway activation, while the color blue indicates predicted pathway inhibition. The z-score statistic is used to determine this, with a threshold of z-scores ≥ 2 and ≤ −2. In gray are the canonical pathways with no predicted z-score, but a significant *p*-value. (**C**) Extracellular matrix muscle proteins. Histogram of common (**left**) and characteristic (**right**) dysregulated extracellular matrix proteins in FSHD mild vs. CTR (black bar) and FSHD severe vs. CTR (gray bar) from the proteomic datasets. (FSHD mild vs. CTR and FSHD severe vs. CTR, ANOVA test and FDR, *p* < 0.05). Proteins are indicated by gene name; the full name is given in the Supplementary Files downloadable at https://doi.org/10.13130/RD_UNIMI/KTS29V. (**D**) Structural and contractile proteins. Histogram of common (left) and characteristic (right) dysregulated structural and contractile proteins in FSHD mild vs. CTR (black bar) and FSHD severe vs. CTR (gray bar) from the proteomic datasets. (FSHD mild vs. CTR and FSHD severe vs. CTR, ANOVA test and FDR, *p* < 0.05). Proteins are indicated by gene name; the full name is given in the Supplementary Files downloadable from https://doi.org/10.13130/RD_UNIMI/KTS29V.

Figure 2A illustrates the characterization of mild and severe patients based on the molecular signature expected for FSHD. In both mild and severe patients, elevated levels of PAX7 and reduced levels of myogenin, particularly pronounced in severe cases, were observed. Panel B presents the results of the canonical pathway analysis conducted using the Ingenuity Pathway Analysis (IPA) software. This analysis identifies key signaling pathways associated with differentially expressed proteins. The results illustrate activated and inhibited canonical pathways in mild and severe patients compared to controls. These pathways are involved in a range of biological processes, including extracellular matrix (ECM) remodeling, metabolism, lipid and cholesterol metabolism, stress response, cell death, and inflammation. The list of proteins involved in each pathway is provided in Supplementary Table S3. IPA analysis revealed activation of the GP6 signaling pathway, including ECM proteins. Panel C displays common and characteristic proteins differentially expressed in mild and severe patients. With regard to common proteins, an increase in collagen alpha-1, alpha-2, and alpha-3 (VI) chain (COL6A1, COL6A2, COL6A3), fibrillin-1 (FBN1), heparan sulfate proteoglycan 2 (HSPG2), laminin subunit gamma-1 (LAMC1), and fibrinogen beta chain (FGB) was observed in both groups. The levels of lumican (LUM), galectin-1 (LGALS1), and calmodulin (CALM1) were decreased in mild and increased in severe patients. Additionally, in severe cases a pronounced accumulation of collagen and molecules promoting ECM interaction and fibrillogenesis was observed. Increased levels of collagen alpha-1 (I) chain, (III) chain, (IV) chain, (V) chain, (XIV) chain, (XV) chain, and (XVIII) chain (COL1A1, COL3A1, COL4A1, COL5A1, COL14A1, COL15A1, COL18A1), collagen alpha-2 (I) chain and (IV) chain (COL1A2, COL4A2), fibulin-1 (FBLN1), laminin subunit alpha-4, alpha-5, beta-1, and beta-2 (LAMA4, LAMA5, LAMB1, LAMB2) and thrombospondin-4 (THBS4) were observed. Molecules promoting ECM interaction, such as nidogen-1 and -2 (NID1, NID2), prolargin (PRELP), and Ras-related protein Rap-1b (RAP1B), and fibril formation, like tenascin-X (TNXB), dermatopontin (DPT), decorin (DCN), fibronectin (FN1), talin-1 (TLN1), and fibrinogen alpha and gamma chain (FGA, FGG) also increased. Only laminin 2 (LAMA2) slightly decreased. Panel D indicates common and characteristic increased proteins in mild and/or severe cases compared to controls. These proteins are involved in cytoskeletal assembly, microtubule organization, actin modulation, intermediate filament organization, and Z-disk and M-line structures, as well as thin and thick filaments. The lists of the total proteins are presented in Supplementary Figures S3 and S4 for mild and severe cases, respectively. In the cytoskeletal region, synaptophysin-like protein 2 (SYPL2), Kelch-like protein 41(KLHL41), and tripartite motif protein (TRIM72) were increased in mild cases and decreased in severe cases. With regard to protein alterations associated with the sarcomere structure, muscle regeneration, and intermediate filaments, the relationship between ECM and myofibers, mediated by focal adhesion kinases (FAKs), was found to be altered, with vinculin (VCL) and vimentin (VIM) increases being more pronounced in severe cases. Pre-laminin (LMNA) was decreased in mild cases and increased in severe cases. Furthermore, several proteins involved in actin modulation were also found to be dysregulated. In particular, WD repeat-containing protein 1 (WDR1) was increased in mild cases, whereas tropomodulin-1 (TMOD1) was increased in severe patients only. Conversely, gelsolin (GLS) and cofilin-1 (CFL1) were increased more significantly in severe cases. Additionally, the dysregulation of giant proteins and Z-disk organization was observed, with an increased and decreased trend for titin (TTN), filamin-C (FLNC), myotilin (MYOT), and alpha-actinin-2 (ACTN2) in mild and severe cases, respectively. Concerning proteins localized at the M-line, a significant increase in myomesin subunits (MYOM1, MYOM2, MYOM3), and four-and-a-half LIM domains 1 (FHL1) was observed in mild cases, in contrast with severe patients. Thick filaments were characterized by increased levels, in mild FSHD, of myosin-binding protein C slow and fast type (MYBPC1, MYBPC2) and of myosin-2 (MYH2), not observed in severe cases where these proteins decreased. With regard to thin filaments, the levels of alpha-actin (ACTA1) and nebulin (NEB) were found to be elevated in mild cases and reduced in patients with severe disease; in contrast, tropomyosin 4 (TPM4) exhibited the opposite behavior. Furthermore, the following proteins were increased in severe cases: myosin-9 (MYH9); myosin-11 (MYH11); myosin light polypeptide 6 (MYL6); myosin regulatory light polypeptide 9 (MYL9); alpha-actinin-1 and 4 (ACTN1, ACTN4), involved in the crosslinking of actin filaments in the cytoskeleton of non-muscle cells; TLN1 and talin 2, regulating the stability of the myotendinous junction; cytoplasmic actin (ACTB); and alpha cardiac muscle actin (ACTC1).

### 3.3. Metabolic Dysregulation

**Figure 3:**
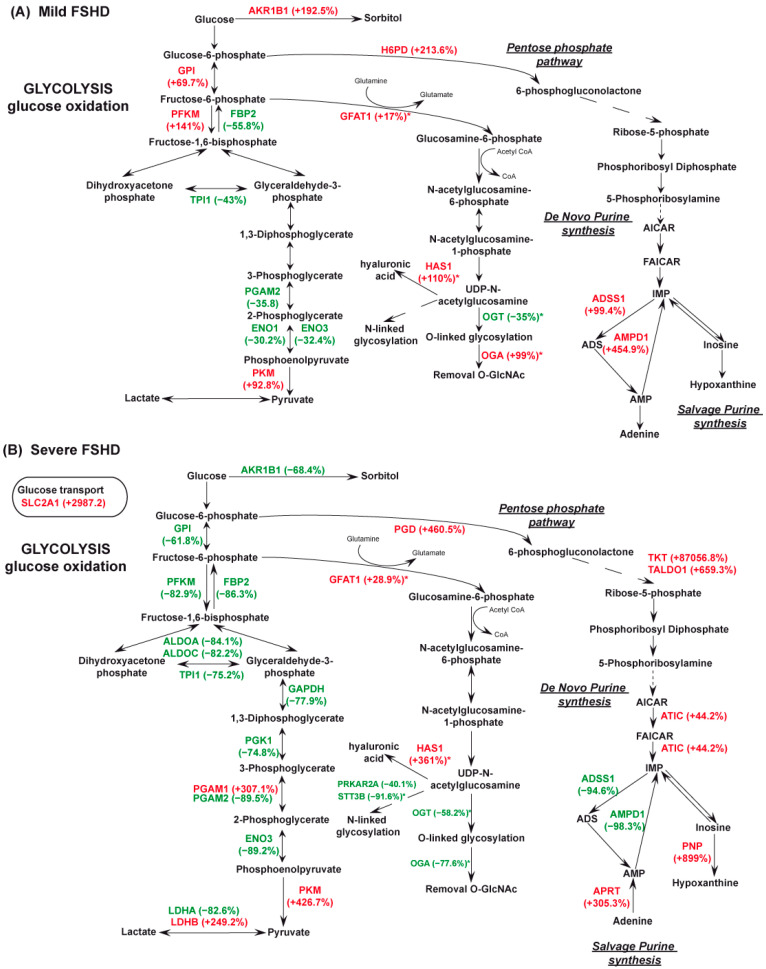
(**A**) Schematic representation of metabolic enzymes dysregulated in FSHD mild vs. CTR. (FSHD mild vs. CTR, ANOVA test and FDR, *p* < 0.05). (**B**) Schematic representation of metabolic enzymes dysregulated in FSHD severe vs. CTR. (FSHD severe vs. CTR, ANOVA test and FDR, *p* < 0.05). * = results obtained by immunoblotting. Green and red colors indicate statistically significant decreases or increases in protein abundance from proteomics datasets, expressed as a % fold change. Proteins are indicated by gene name; the full name is given in the Supplementary Files downloadable from https://doi.org/10.13130/RD_UNIMI/KTS29V.

**Figure 4:**
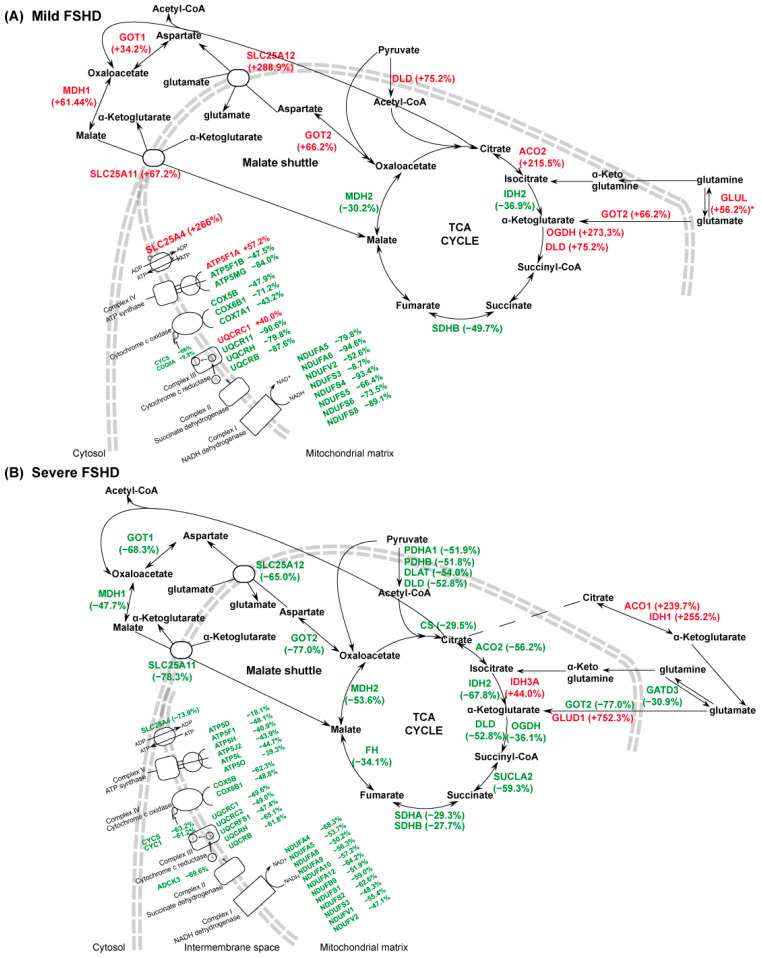
(**A**) Schematic representation of metabolic enzymes dysregulated in FSHD mild vs. CTR. (FSHD mild vs. CTR, ANOVA test and FDR, *p* < 0.05). (**B**) Schematic representation of metabolic enzymes dysregulated in FSHD severe vs. CTR. (FSHD severe vs. CTR, ANOVA test and FDR, *p* < 0.05). * = results obtained by immunoblotting. Green and red colors indicate statistically significant decreases or increases in protein abundance from proteomics datasets, expressed as a % of fold change. Proteins are indicated by gene name; the full name is given in the Supplementary Files downloadable from https://doi.org/10.13130/RD_UNIMI/KTS29V.

In FSHD patients, IPA analysis suggested metabolic dysregulation at the glycolytic, TCA cycle, and respiratory chain levels. This is illustrated in Figure 3 and Figure 4.

*Glycolysis*. In mild cases, a downregulation of fructose-bisphosphatase 2 (FBP2) and an upregulation of ATP-dependent 6-phosphofructokinase muscle type (PFKM), and glucose-6-phosphate isomerase (GPI) were observed; in severe cases, these enzymes decreased. Furthermore, increased levels of aldo-keto reductase 1B (AKR1B1), promoting NADPH production, characterized mild patients. Differential changes in glycolytic enzymes between mild and severe patients compared to controls indicated an inhibition of glycolysis. In mild cases, a decrease in triosephosphate isomerase (TPI1), phosphoglycerate mutase 2 (PGAM2), alpha-enolase (ENO1), and enolase 3 (ENO3) was observed; whereas pyruvate kinase (PKM) increased. In severe cases, decreased levels of fructose-bisphosphate aldolase A and C (ALDOA, ALDOC), TPI1, glyceraldehyde-3-phosphate dehydrogenase (GAPDH), phosphoglycerate kinase 1 (PGK1), PGAM2, ENO3, and L-lactate dehydrogenase A chain (LDHA) were observed. However, increased levels of phosphoglycerate mutase 1 (PGAM1), PKM, L-lactate dehydrogenase B chain (LDHB), and the glucose transporter SLC2A1 (solute carrier family 2 member 1) were observed (Figure 3A, B).

*Pentose phosphate pathway (PPP) and purine metabolism*. In the pentose phosphate pathway (PPP), mild FSHD patients exhibited increased levels of hexose-6-phosphate dehydrogenase (H6PD), a significant source of reducing power and metabolic intermediates that can be used as an alternative to glycolysis. Mild cases show increased levels of two enzymes involved in IMP synthesis, AMP deaminase 1 (AMPD1) and adenyl succinate synthase (ADSS1). In contrast, severe patients demonstrated increased levels of phosphogluconate dehydrogenase (PGD), the second dehydrogenase of the PPP, as well as increased levels of transketolase (TKT) and transaldolase (TALDO), indicating activation of the reductive PPP. Decreased levels of AMPD1 and ADSS1 are observed in severe cases. Furthermore, adenine phosphoribosyl transferase (APRT), purine nucleoside phosphorylase (PNP), and the bifunctional purine biosynthesis enzyme (ATIC) show increased levels, suggesting an energetically less costly salvage formation of AMP and enhanced purine metabolism. As shown in Figure 3A,B for mild and severe patients, respectively, this differential expression profile in PPP enzymes is evident.

*TCA Cycle*. In mild patients, aconitate hydratase (ACO2), 2-oxoglutarate dehydrogenase (OGDH), and dihydrolipoyl dehydrogenase (DLD) increased, whereas mitochondrial NADP+-dependent isocitrate dehydrogenase (IDH2), the succinate dehydrogenase [ubiquinone] iron-sulfur subunit (SDHB), and malate dehydrogenase 2 (MDH2) decreased. With regard to the malate/aspartate shuttle, increased levels of cytoplasmic and mitochondrial aspartate aminotransferase (GOT1, GOT2), cytosolic malate dehydrogenase (MDH1), mitochondrial 2-oxoglutarate/malate carrier protein (SLC25A11), and calcium-binding mitochondrial carrier protein Aralar1 (SLC25A12) were observed (Figure 4A). In patients with severe disease, increased levels of cytosolic aconitate hydratase (ACO1), cytoplasmic NADP+-dependent isocitrate dehydrogenase (IDH1), mitochondrial glutamate dehydrogenase 1 (GLUD1), and mitochondrial isocitrate dehydrogenase [NAD] subunit alpha (IDH3A) were observed. In contrast, pyruvate dehydrogenase E1 component subunit alpha and beta (PDHA1, PDHB), dihydrolipoyllysine-residue acetyltransferase (DLAT), citrate synthase (CS), ACO2, IDH2, OGDH, DLD, succinyl-CoA ligase [ADP-forming] subunit beta (SUCLA2), succinate dehydrogenase [ubiquinone] flavoprotein subunit (SDHA), SDHB, fumarate hydratase (FH), MDH2, GOT1, GOT2, MDH1, SLC25A11, SLC25A12, and glutamine amidotransferase-like class 1 domain-containing protein 3 (GATD3) decreased. Collectively, these results indicated the preservation of the oxidative TCA cycle in mild cases and the use of the reductive TCA cycle in severe cases (Figure 4B).

*Respiratory Chain*. Mild patients showed decreased levels of multiple respiratory chain components, while severe patients displayed an overall decrement of OXPHOS components in the respiratory chain (Figure 4). In mild cases, NADH dehydrogenase [ubiquinone] 1 alpha subcomplex subunit 5 and 6 (NDUFA5, NDUFA6), NADH dehydrogenase [ubiquinone] flavoprotein 2, (NDUFV2), NADH dehydrogenase [ubiquinone] iron-sulfur protein 3, 4, 5, 6, and 8 (NDUFS3, NDUFS4, NDUFS5, NDUFS6, NDUFS8), cytochrome b-c1 complex subunit 6, 7, and 10 (UQCRH, UQCRB, UQCR11), cytochrome c oxidase subunit 5B, 6B1, and 7A1 (COX5B, COX6B1, COX7A1), ATP synthase F1 subunit beta and membrane subunit g (ATP5F1B, ATP5MG), cytochrome C (CYCS), and atypical kinase COQ8A (COQ8A) decreased. In contrast, cytochrome B-C1 complex subunit 1 (UQCRC1), ATP synthase F1 subunit alpha (ATP5F1A), and ADP/ATP translocase 1 (SLC25A4) increased.

Patients with severe disease were characterized by reduced levels of several complex I, II, III, and V subunits and of SLC25A4. These findings suggest a consistent impairment of the respiratory chain and metabolic deficiency in severe patients, whereas mild patients showed signs of compensation not observed in severe patients. In severe patients, the impairment of the mitochondrial respiratory chain and metabolic deficiency appear to be more pronounced.

### 3.4. Lipid Metabolism

**Figure 5:**
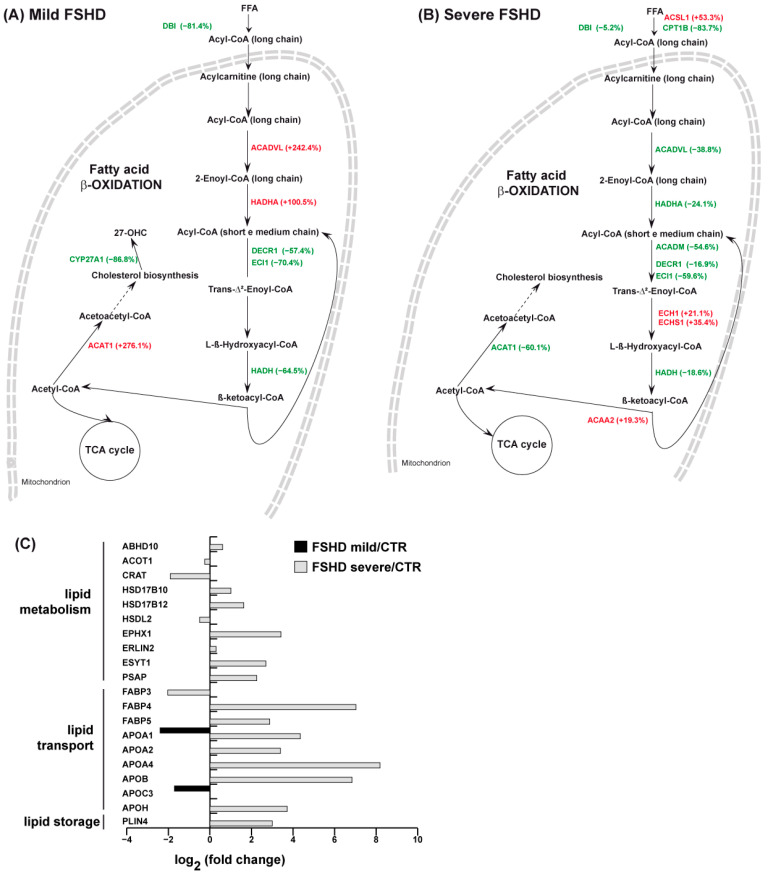
(**A**) Schematic representation of lipid enzyme dysregulation in FSHD mild vs. CTR. (FSHD mild vs. CTR, ANOVA test and FDR, *p* < 0.05). (**B**) Schematic representation of lipid enzymes’ dysregulation in FSHD severe vs. CTR. (FSHD severe vs. CTR, ANOVA test and FDR, *p* < 0.05). Green and red colors indicate statistically significant decreases or increases in protein abundance from proteomics datasets, expressed as a % fold change. (**C**) Histograms of dysregulated proteins involved in lipid transport and storage in FSHD mild vs. CTR (black bar) and FSHD severe vs. CTR (gray bar) from the proteomic datasets (FSHD mild vs. CTR and FSHD severe vs. CTR, ANOVA test and FDR, *p* < 0.05). Proteins are indicated by gene name; the full name is given in the Supplementary Files downloadable from https://doi.org/10.13130/RD_UNIMI/KTS29V.

IPA analysis indicated dysregulation of the DHCR24 signaling pathway and LXR/RXR activation in severe patients. The results are shown in Figure 5A, and 5B, for mild and severe patients, respectively. Mild patients were characterized by a decrease in acyl CoA-binding protein (DBI) and of enzymes involved in the processing of medium- and short-chain acyl-CoA, including 2,4-dienoyl CoA reductase (DECR1), enoyl CoA delta isomerase 1 (ECI1), and hydroxyacyl-coenzyme A dehydrogenase (HADH). In contrast, increased levels of enzymes involved in acyl-CoA long-chain processing including very-long-chain specific acyl-CoA dehydrogenase (ACADVL) and trifunctional enzyme subunit alpha (HADHA) were observed. Cholesterol synthesis was promoted by an increased level of acetyl-CoA acetyltransferase (ACAT1); however, the enzyme controlling cholesterol homeostasis, sterol 26-hydroxylase (CYP27A1), decreased. Severe patients were characterized by decreased levels of DBI and carnitine O-palmitoyltransferase 1 (CPT1B), the transporter of fatty acids from the cytosol to mitochondria, and increased levels of long-chain-fatty-acid-CoA ligase 1 (ACSL1). Enzymes involved in fatty acid beta-oxidation are impaired; particularly, decreased levels of ACADVL, HADHA, medium-chain-specific acyl-CoA dehydrogenase (ACADM), DECR1, ECI1, and HADH were observed. In contrast, delta(3,5)-Delta(2,4)-dienoyl-CoA isomerase (ECH1), enoyl-CoA hydratase (ECHS1), and 3-ketoacyl-CoA thiolase (ACAA2) increased. Furthermore, ACAT1, involved in cholesterol synthesis, was also decreased. Collectively, these results suggest a partial activation of fatty acid beta-oxidation in mild cases not observed in severe cases, in which the activation of the reductive TCA cycle leads to the rerouting of citrate to fatty acid synthesis, resulting in cytosolic lipid accumulation. Figure 5C indicates several proteins involved in lipid metabolism, transport, and storage increased in severe cases only, including 3-hydroxyacyl-CoA dehydrogenase type-2 (HSD17B10), very-long-chain 3-oxoacyl-CoA reductase (HSD17B12), fatty acid-binding protein (FABP4, FABP5), Apolipoprotein A-II, A-IV, B-100, and H (APOA2, APOA4, APOB, APOH), and perilipin-4 (PLIN4), further supporting the impairment of lipid metabolism and lipotoxicity.

### 3.5. NADP^+^/NADPH and NAD^+^/NADH Imbalance

**Figure 6:**
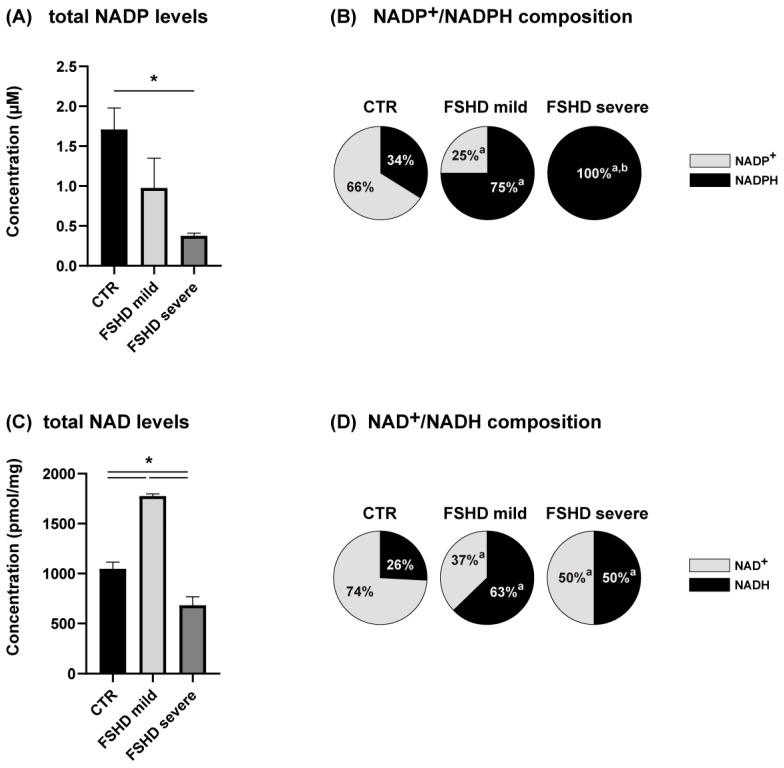
(**A**) Bar chart showing nicotinamide adenine dinucleotide phosphate cofactor levels in controls (CTR, black bar) and in mild (light gray bar) and severe (dark gray bar) FSHD patients. * = significant changes (ANOVA + Tukey, n = 2, *p* < 0.05); (**B**) Pie chart indicating the % of oxidized (NADP^+^, gray) and reduced (NADPH, black) nicotinamide adenine dinucleotide phosphate cofactor in CTR, FSHD mild, and FSHD severe cases. a = significant variation compared to CTR, b = significant variation compared to FSHD mild (ANOVA + Tukey, n = 2, *p* < 0.01). (**C**) Bar chart showing nicotinamide adenine dinucleotide cofactor levels in CTR (black bar) and in mild (light gray bar) and severe (dark gray bar) FSHD patients. * = significant changes (ANOVA + Tukey, n = 2, *p* < 0.05); (**D**) Pie chart indicating the percentage of oxidized (NAD^+^, gray) and reduced (NADH, black) nicotinamide adenine dinucleotide cofactor in each experimental group. a = significant variation compared to CTR (ANOVA + Tukey, n = 2, *p* < 0.01).

Upon nutrient stress related to the glycolytic pathway and electron transport deficiency compromising ATP production, muscle tissue becomes vulnerable to oxidoreductive stress. The levels of cellular NAD(H)/NADP(H) are essential for maintaining redox homeostasis. Decreases in these redox couples can lead to oxidative or reductive stress, depending on the redox ratio of each. To evaluate the compensatory metabolic mechanisms adopted in mild cases and absent in severe cases, the total pool of redox cofactors and the levels of their reduced and oxidized forms were assessed as shown in Figure 6. Concerning the NADP pool, a decreased level was observed in mild cases, although not statistically significant compared to controls (Figure 6A), whereas in severe cases the total pool was significantly decreased. In mild cases, a switch toward the reduced form is observed with an increase in NADPH levels compared to NADP^+^ (Figure 6B). In severe cases, lower levels of NADP pool were completely converted to NADPH, and NADP^+^ was below the limit of our test detectability.

Regarding the total pool of NAD (Figure 6C), increased levels were observed in mild cases and decreased levels in severe cases compared to controls. In both mild and severe patients, the levels of oxidized and reduced forms (NAD^+^/NADH) indicated a switch toward NADH compared to controls, with no statistically significant differences between mild and severe patients (Figure 6D), indicating that the imbalance of the NAD pool is a common feature in both mild and severe cases.

### 3.6. Stress Response and Hexosamine Biosynthetic Pathway (HBP)

**Figure 7:**
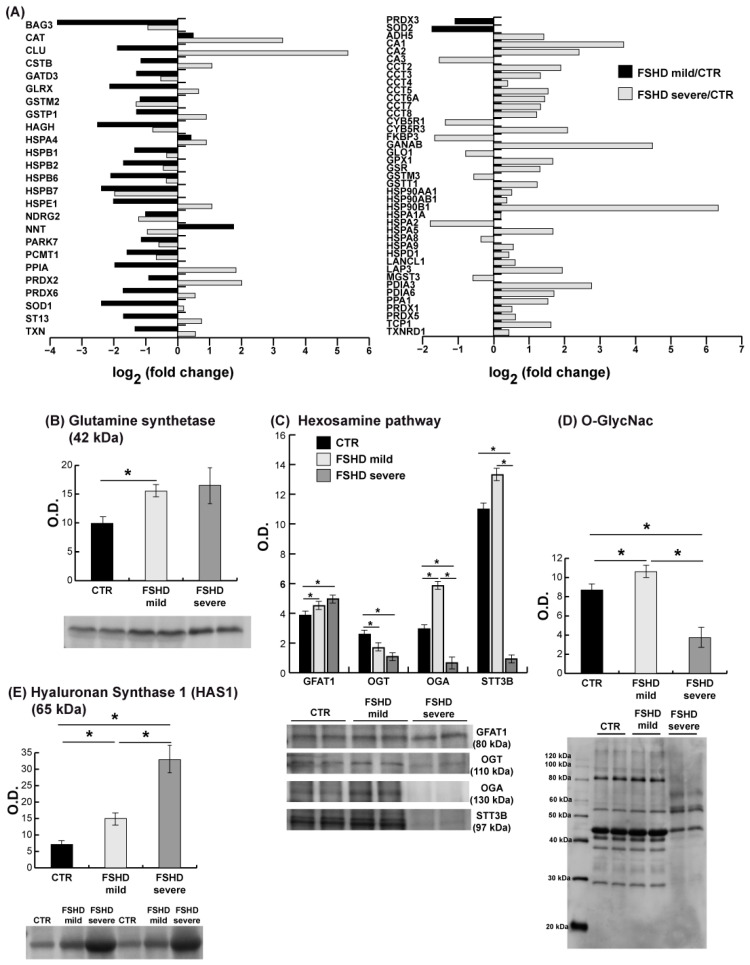
Stress response proteins and the hexosamine biosynthetic pathway. (**A**) Histogram of common (left) and characteristic (right) dysregulated proteins’ levels of stress response in FSHD mild vs. CTR (black bar) and FSHD severe vs. CTR (gray bar) from the proteomic datasets. (FSHD mild vs. CTR and FSHD severe vs. CTR, ANOVA test and FDR, *p* < 0.05). Proteins are indicated by gene name, the full name is given in the Supplementary Files downloadable from https://doi.org/10.13130/RD_UNIMI/KTS29V. (**B**–**E**) Representative bar graph (means ± SD) and immunoblot images of glutamine synthetase, hexosamine pathway (GFAT1, OGT, OGA, STT3B), O-GlcNac and hyaluronan synthase 1 (HAS1) (n = 2; mean ± SD; Student’s *t*-test, *p* < 0.05) in healthy controls (CTR, black bars) and FSHD mild and severe patients (light and dark gray bars). Data were normalized against the total amount of loaded proteins stained with Sypro Ruby. O.D. = optical density. * = statistically significant. Full-length images are available in Supplementary Figure S5.

The stress response proteins and enzyme levels regulating the hexosamine biosynthetic pathway are illustrated in Figure 7.

*Stress response.* Mild cases are characterized by increased levels of NAD(P) transhydrogenase mitochondrial (NNT), catalase (CAT), and heat shock 70 kDa protein 4 (HSPA4). However, the majority of cytoplasmic and mitochondrial proteins involved in the stress response showed decreased levels. Specifically, proteins such as BAG family molecular chaperone regulator 3 (BAG3), heat shock proteins (HSPB1, HSPB2, HSPB6, HSPB7), 10 kDa heat shock protein (HSPE1), clusterin (CLU), hsc70-interacting protein (ST13), cystatin-B (CSTB), protein NDRG2, and protein deglycase DJ-1 (PARK7) decreased. Glutathione handling also decreased, as evidenced by the decrease in mitochondrial hydroxyacylglutathione hydrolase, (HAGH), cytosolic glutaredoxin-1 (GLRX), cytosolic glutathione S-transferase P (GSTP1), cytosolic glutathione S-transferase mu 2 (GSTM2), and of mitochondrial glutamine aminotransferase class 1 domain containing 3 (GATD3). Furthermore, several other proteins involved in the stress response showed decreased levels, including superoxide dismutase [Cu-Zn] (SOD1), superoxide dismutase [Mn], mitochondrial (SOD2), protein-L-isoaspartate (D-aspartate) O-methyltransferase (PCMT1), peptidyl-prolyl cis-trans isomerase A (PPIA), peroxiredoxin 2 (PRDX2), peroxiredoxin 6 (PRDX6), thioredoxin (TXN), and thioredoxin-dependent peroxide reductase, mitochondrial (PRDX3). In severe patients, 44 stress-related proteins showed increased levels, while 20 exhibited decreased levels. Notably, several proteins exhibit opposite trends between mild and severe patients, including CLU, CSTB, GLRX, GSTP1, HSPE1, NNT, PPIA, PRDX2, PRDX6, SOD1, and ST13. Conversely, proteins such as BAG3, CAT, GATD3, GSTM2, HAGH, HSPA4, HSPB1, HSPB2, HSPB6, HSPB7, NDRG2, PARK7, and PCMT1 exhibit similar trends. Furthermore, in severe patients, mitochondrial proteins such as NADH-cytochrome b5 reductase 1 and 3 (CYB5R1, CYB5R3), relevant for redox equilibrium, decreased and increased, respectively. In severe patients, HSPA5 (GPR78) increased, supporting the NRF2-mediated oxidative stress response, as highlighted by IPA analysis. Collectively, these results, supported by the NAD(H)/NADP(H) cellular levels, indicate oxidative stress in mild cases and suggest reductive stress in severe cases.

*The hexosamine biosynthetic pathway*, which utilizes glutamine, is involved in the stress response and unfolded protein response. The results in Figure 7B indicated an increase in glutamine synthetase (GLUL) in both mild and severe cases compared to CTR. Immunoblotting of muscle extracts of enzymes regulating HBP indicated increased levels of glutamine-fructose-6-phosphate dehydrogenase (GFAT1) in both mild and severe cases compared to CTR (Figure 7C). In mild FSHD patients, O-GlcNAcase (OGA), responsible for protein O-deglycosylation, increased, whereas protein O-glycosylation, mediated by O-GlcNAc transferase (OGT), decreased. N-glycosylation, mediated by STT3B, increased, although this was not statistically significant. In severe cases, OGT, OGA and STT3B decreased compared to both CTR and FSHD mild cases. However, immunoblotting of the total O-glycosylated proteins (Figure 7D) indicated that O-glycosylated protein levels were increased in mild cases and decreased in severe cases, suggesting that mild cases could better cope with ER stress by reducing the unfolded protein response (UPR), thereby promoting survival. Decreased levels of enzymes regulating N- and O-glycosylation promoted hyaluronic acid synthesis by hyaluronan synthase 1 (HAS1), as indicated in Figure 7E, through the use of UDP-GlcNAc, which was not consumed by STT3B, OGT, and OGA in severe cases. A slight increase was also observed in mild cases, likely due to decreased levels of OGT.

### 3.7. Inflammation and Immune Response

**Figure 8:**
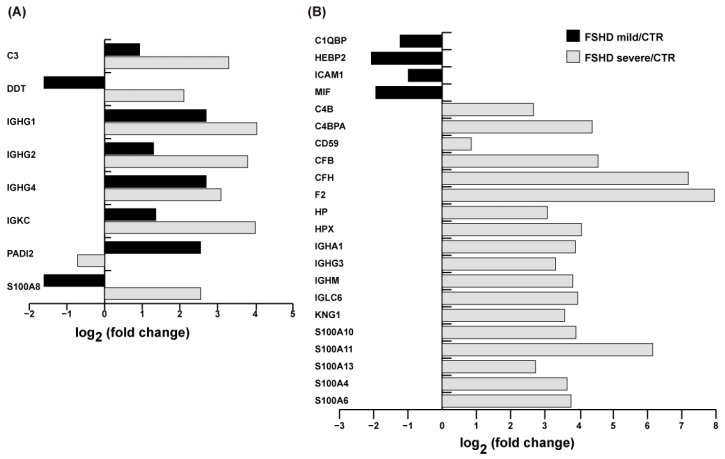
Inflammation and immune response proteins. (**A**) Histograms of common dysregulated proteins levels of inflammation and immune response in FSHD mild vs. CTR (black bar) and FSHD severe vs. CTR (gray bar) from the proteomic datasets. (FSHD mild vs. CTR and FSHD severe vs. CTR, ANOVA test and FDR, *p* < 0.05). (**B**) Histogram of dysregulated proteins levels of inflammation and the immune response in FSHD mild vs. CTR and in FSHD severe vs. CTR (gray bar) from the proteomic datasets. Proteins are indicated by gene name; the full name is given in the Supplementary Tables downloadable from https://doi.org/10.13130/RD_UNIMI/KTS29V.

The dysregulation of proteins involved in inflammation and immune response distinguishes mild from severe patients. Figure 8 displays the common and characteristic proteins differentially expressed in both mild and severe patients. In mild cases, a few proteins showed increased levels, such as peptidyl arginine deiminase 2 (PADI2), Ig gamma C region 1, 2, and 4 chain (IGHG1, IGHG2, IGHG4), Ig kappa chain C region (IGKC), and complement C3 (C3). Conversely, proteins such as heme-binding protein 2 (HEBP2), macrophage migration inhibitory factor (MIF), D-dopachrome decarboxylase (DDT), protein S100-A8 (S100A8), complement component 1 Q subcomponent-binding protein, mitochondrial (C1QBP), and intercellular adhesion molecule 1 (ICAM1) decreased. In severe patients, C3, DDT, IGHG1, IGHG2, IGHG4, IGKC, and S100A8 were increased, while PADI2 was decreased. Furthermore, severe patients exhibited increased levels of proteins absent in mild patients. Among these proteins were Ig alpha-1 chain C region (IGHA1), Ig gamma-3 chain C region (IGHG3), Ig mu chain C region (IGHM), and Ig lambda-6 chain C region (IGLC6), which are located in the major histocompatibility complex (MHC) and are involved in the activation of the immune response. In addition, the following were also upregulated, only in severe cases: kininogen-1 (KNG1), inhibitor of proteases, prothrombin (F2), haptoglobin (HP), hemopexin (HPX), complement C4-B (C4B), C4b-binding protein alpha chain (C4BPA), complement factor B and H (CFB, CFH), CD59 glycoprotein (CD59), and proteins promoting calcium binding and involved in the regulation of the cell cycle, differentiation, and inflammatory response, such as protein S100 (S100A10, S100A11, S100A13, S100A4, S100A6).

### 3.8. Validation of Proteomic Data by Immunoblotting and Quantitative PCR Analysis

In response to metabolic impairment induced by decreased glycolytic activity, muscle tissues may engage various catabolic processes to generate essential building blocks and ATP for biosynthesis. Glucose deprivation can stimulate proteasomal degradation, activate lysosomes, and induce autophagy.

**Figure 9:**
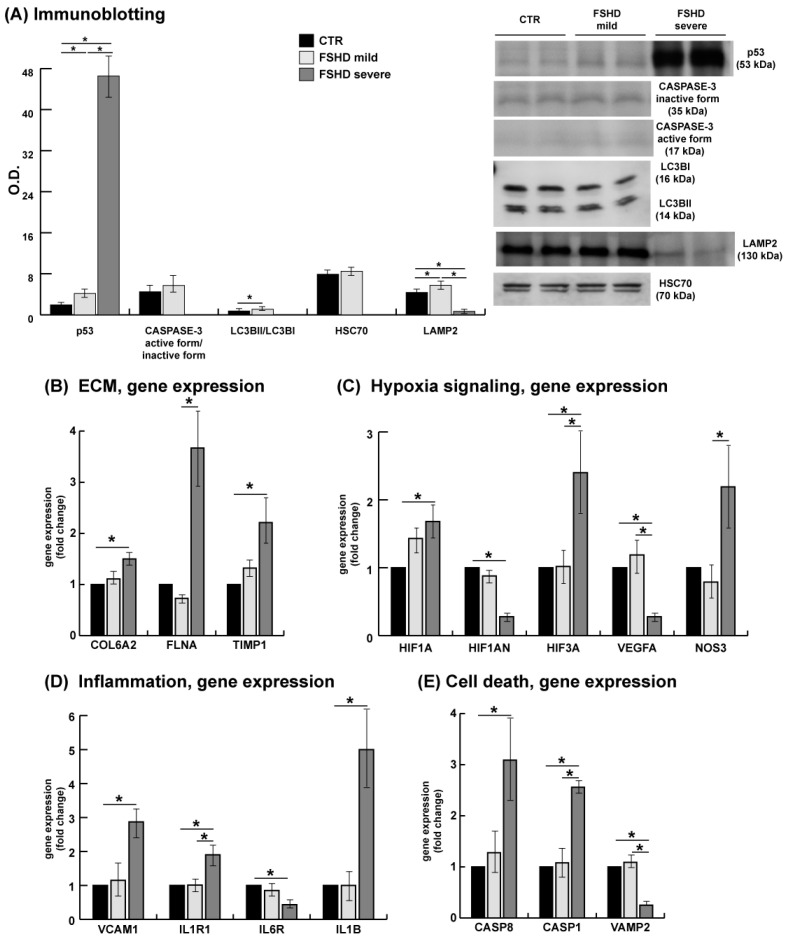
(**A**) Representative bar graph (means ± SD) and immunoblot images of p53, CASPASE-3, LC3BII/LC3BI, HSC70, and lysosome-associated membrane protein 2 (LAMP2) in healthy controls (CTR, black bars) and mild and severe FSHD patients (gray bars) (n = 2; mean ± SD; Student’s *t*-test, *p* < 0.05). Data were normalized against the total amount of loaded proteins stained with Sypro Ruby. O.D. = optical density. Full-length images are available in Supplementary Figure S5. (**B**–**E**) Validation of proteomic data by quantitative PCR analysis performed in the OpenArray^®^ QuantStudio 12K Flex Real-Time PCR System, using the commercial probes shown in Supplementary Table S2. The GAPDH, ACTB, and 18S genes were included in the OpenArray^®^ chip and used as housekeeping endogenous control genes. Each analysis was conducted in duplicate. * = statistically significant.

To investigate these processes in FSHD patients, targets suggested by proteomic results were examined through immunoblotting. The results revealed an increase in p53, particularly pronounced in severe cases. Conversely, the levels of LC3BII/LC3BI and LAMP2 increased in mild patients, indicating the activation of autophagy and lysosomal degradation. In severe cases, the LAMP2 level decreased (see Figure 9A).

These findings suggest that compensatory mechanisms activated in mild patients include modulation of the HBP to control the stress response, overproduction of NNT to sustain mitochondrial NADPH production, and the activation of autophagy and lysosomal degradation to support the biosynthesis of essential building blocks.

Furthermore, the negative outcomes in a selected group of genes highlighted in previous FSHD studies were investigated in mild and severe patients, as listed in Supplementary Table S3, including genes involved in ECM, hypoxia signaling, inflammation, and apoptosis (Figure 9). In relation to ECM, the results confirmed a significant increase in gene expression in severe patients compared to controls of COL6A2 and metallopeptidase inhibitor 1 (TIMP1). Furthermore, filamin A (FLNA) was significantly increased in severe compared to mild cases (Figure 9A). Concerning hypoxia signaling, a significant increase in gene expression was observed in severe cases compared to CTR for hypoxia inducible factor 1 and 3 subunit alpha (HIF1A, HIF3A). Nitric oxide synthase 3 (NOS3) and HIF3A were significant upregulated in severe compared to mild cases. HIF1A inhibitor (HIF1AN) was significantly decreased in severe cases compared to controls; the level of vascular endothelial growth factor A (VEGFA) was found to be reduced in severe cases when compared to both control subjects and mild cases (Figure 9B). The inflammation observed in the proteomic analysis was confirmed by a significant increase in the gene expression of vascular cell adhesion molecule 1 (VCAM1), interleukin 1 receptor type 1 (IL1R1), and interleukin 1 beta (IL1B), and decreased levels of the interleukin 6 receptor (IL6R) in severe patients when compared to control subjects. Regarding the processes of apoptosis and cell death, the transcript levels of caspase 8 and 1 (CASP8, CASP1) significantly increased in severe cases compared to control subjects, whereas vesicle associated membrane protein 2 (VAMP2) decreased in severe cases compared to mild and control subjects. Unfortunately, other confirmatory tests particularly on severe patients were limited due to restricted sample availability.

## 4. Discussion

FSHD is still an incurable disease with multifaceted etiology and new treatments are needed, including customized approaches that depend on disease severity. With the aim of gaining deeper insight into the biochemical pathways responsible for muscle degeneration in FSHD, we performed a muscle proteome analysis of mild and severe patients classified based on the Ricci score and on 2D-DIGE PCA followed by LC MS/MS label-free proteomic analysis to highlight the molecular nodes associated with lower muscle impairment in mild cases. PCA from 2D-DIGE proteomic analysis indicates no difference in the proteomic pattern in control subjects, whereas, according to the Ricci score, a clusterization of mild versus severe patients was observed. The molecular markers typically associated with FSHD are dysregulated in both mild and severe patients [8,47], as indicated in Figure 2A. Utilizing IPA canonical pathway analysis, we identified both similarities and discrepancies between mild and severe cases. While several pathways showed activation/inhibition in both mild and severe FSHD, the prediction was notably stronger in severe cases, evidenced by higher z-scores and *p*-values.

In mild cases, certain pathways did not reach significance thresholds (Figure 2B); however, the analysis can be indicative of nodes involved in the disease severity. Pathways associated with GP6 signaling and ECM dysregulation showed a significant *p*-value in mild cases, while both a significant *p*-value and z-score characterized severe FSHD. The ECM signature indicated an abnormal increase in the collagen (VI) chain, as previously described [14], aligning with a similar pattern observed in other conditions where chromatin relaxation and CBP/p300 activation were present [45,48]. ECM molecular targets of DUX4 were confirmed in both mild and severe cases (COL6A1, COL6A2, COL6A3, FBN1, FGB, HSPG2, and LAMC1) [14,49]. In mild cases, ECM dysregulation is associated with decreased levels of calmodulin, galectin, and lumican, which are involved in muscle homeostasis and repair and could be possible targets for FSHD treatments [50,51,52,53,54].

Severe patients showed a general increase in collagens and molecules involved in the laminin–dystroglycan and fibronectin–integrin (α7β1) axes, as well in the connection between laminin and collagen VI. Increased levels of proteins involved in matrix assembly and cell matrix interactions promoting structural changes and fibril formation [55] are confirmed by increased levels of the metallopeptidase inhibitor 1 (TIMP1) and filamin A, and of COL6A2 mRNA transcripts (Figure 9A). ECM dynamics contribute to muscle impairment in both mild and severe FSHD. Collagen VI overexpression and chromatin relaxation due to histone modifications drive inflammation and fibrosis, further exacerbating muscle weakness. Profibrotic stimuli promoting ECM synthesis rely on metabolic reprogramming with glutamine production that supports collagen synthesis, as observed in cancer cells [56].

ECM changes impact muscle at structural and contractile protein levels [57] (Figure 2D and Supplementary Figures S3 and S4), as confirmed by variation in many proteins between mild and severe cases. In mild cases, increased levels of proteins in the sarcomeric structure, actin modulation, thick and thin filament, and M-line suggest a partial preservation of muscle function [58], which is not observed in severe cases. In severe patients, dysregulation of vinculin, filamin-C, cofilin-1, myotilin, and sarcomeric actinins are associated with lower myoblast fusion and differentiation [59,60,61,62] and NMJ integrity [63,64]. Increased levels of COL4A1, COL4A2, LAMA2, LAMB1, and LAMA5 involved in basal lamina composition were observed [5]. Notably, non-muscle ACTN1 and ACTN4 levels were increased. These have recently been associated with Cullin-3 inhibition, affecting ubiquitin-proteasome signaling, promoting the activation of NRF2, inhibition of myogenesis, and myofiber hypotrophy [65,66], suggesting ACTN1 and ACTN4 as targets for further studies.

Muscle is a high metabolic tissue, and metabolic dysregulation impacts muscle function. IPA canonical pathway analysis indicated that metabolism is differently influenced between mild and severe patients, and structural changes are closely associated with metabolic alterations.

The focus of this paper is on metabolic differences characterizing severe patients compared to mild. Metabolic alterations were apparent in both conditions, with a decline in glycolysis, TCA cycle, and oxidative phosphorylation, promoting mitochondrial dysfunction [67,68], partially offset by metabolic adaptations in mild cases.

Our proteomic data in mild patients revealed changes in the levels of key enzymes and transporters involved in energy homeostasis. Glycolysis was reduced, with glucose redirected toward polyol synthesis and the oxidative pentose phosphate pathway (PPP) for ribose 5-phosphate (R5P) production. Polyol pathway activity was elevated, as indicated by increased levels of AKR1B1, leading to the production of sorbitol and fructose. Accumulation of sorbitol and fructose has been observed in ALS patients [69] in dermatomyositis and in DMD animal models [70,71] suggesting a possible contribution to muscle atrophy in FSHD as well.

Additionally, glucose-6-phosphate was channeled toward the oxidative PPP for the production of ribose 5-phosphate, which is essential for the synthesis of nucleotides, RNA, and DNA, as well as for generating cytosolic NADPH [72]. Pyruvate production was supported by the activation of PKM, which promotes the malate/aspartate shuttle [73].

Analysis of samples from mild patients clearly indicate a partial reprogramming of mitochondrial metabolism toward an increased activity of the malate/aspartate shuttle. However, this is not very well compensated in mitochondria since the downregulation of TCA dehydrogenases (as MDH2) coupled to reduced respiratory chain activity probably result in intramitochondrial NADH accumulation. In this case, pyruvate might contribute to mitochondrial oxaloacetate through pyruvate carboxylase [74]. Glutamine is converted into glutamate and alpha-ketoglutarate, indicating an anaplerotic use of the TCA cycle [75,76,77]. This oxidative influx from glutamine becomes a source of ATP [78]. Oxidation of succinyl-CoA to succinate generates substrate-level production of ATP/GTP, becoming an independent source of high-energy phosphate not related to OXPHOS or glycolysis [79]. Overall, our data suggest that through glutamine anaplerosis of the TCA cycle and NNT-mediated mitochondrial NADPH production, promoted by NADH accumulation, the muscle of mild cases can sustain energy-generating pathways as confirmed by the increase in reduced redox cofactors (Figure 6). NADPH protects against oxidative stress both directly, by neutralizing reactive oxygen species, and indirectly, by regenerating reduced glutathione (GSH) from its oxidized form (GSSG) [80].

In response to metabolic impairment induced by decreased glycolytic activity, muscle may engage various catabolic processes to generate essential building blocks and ATP for biosynthesis. Glucose deprivation can stimulate proteasomal degradation, activate lysosomes, and induce autophagy [81,82,83]. These findings suggest that compensatory mechanisms activated in mild patients include the activation of the PPP for the synthesis of building blocks, modulation of the HBP to control the stress response [84], increased production of NNT to support mitochondrial NADPH production [85], and the activation of autophagy and lysosomal degradation to supply essential biosynthetic components [86].

Additionally, in mild cases, glutamine is utilized to control the endoplasmic reticulum (ER) stress, sustaining autophagy, cell anabolism and survival [76,87,88]. These adaptations can contribute to maintaining muscle function by compensating for impaired mitochondrial respiratory chain activity.

Unfortunately, the assessment of mitochondrial respiration was hampered by the lack of tissue, so we based our considerations on the levels of molecules provided by proteomic results indicating a downregulation of more than 50% in all complexes, but also highlighted molecules at variance between mild and severe FSHD. In mild cases, the upregulation of a few molecules, including the ATP/ADP transporter SLC25A4 (ANT1), was observed. This appears to be one of the distinguishing elements in our proteomic dataset, with ANT1 levels reduced in severe patients. However, the literature on ANT1 protein and mRNA expression levels in FSHD remains controversial [19,89,90,91,92]. Our data suggest a possible association between ANT1 expression and disease severity, though further studies are needed to clarify this, particularly in cell models grown in low-nutrient media that mimic the metabolic conditions seen in mild and severe patients. Proteomic analysis of severe patients indicates elevated levels of the GLUT1 transporter, inhibition of glycolysis, and unchanged levels of enzymes regulating the polyol pathway. Consequently, cytosolic NADPH production and the synthesis of building blocks rely solely on the reductive PPP. Furthermore, their metabolism is significantly impaired, relying solely on glutamine, which enters the mitochondria and, via GLUD1, is converted into α-ketoglutarate [93]. The impairment of mitochondrial metabolism, likely coupled with a loss of mitochondrial integrity and membrane potential, is inferred by the upregulation of IDH1 and ACO1. These upregulations suggest that citrate is probably synthetized solely through the reductive carboxylation of α-ketoglutarate. This activity counteracts NADP+/NADPH imbalance by oxidizing NADPH to NADP+ in the cytosol [94]. LDHB is also used in reverse to generate pyruvate to sustain mitochondrial function [95]. Following targeted and untargeted metabolic tracing in cells with complete OXPHOS impairment, it has been demonstrated that reductive glutamine metabolism is mainly directed toward anabolic processes promoting acetylation and lipid biosynthesis as occurs in severe patients [76].

Increased lipid storage has been linked to oxidative stress, promoting ROS generation and inflammation in FSHD [96]. Our proteomic results highlight significant changes in several proteins involved in lipid synthesis and metabolism in severe patients, such as an increase in ACSL1 and a decrease in CPT1B, along with an increase in fatty acid binding proteins, apolipoproteins, and perilipins involved in lipid vesicle formation and maturation [97]. This indicates an anabolic reprogramming of glutamine metabolism, confirming a decrease in lipid processing and induction of de novo lipogenesis in severe patients. However, the oxidative stress induced by lipid peroxidation may contribute to the accumulation of damaged proteins, promoting apoptosis and inflammation [98,99].

Both mild and severe patients displayed modulation in key regulators of inflammation and oxidative stress, particularly regarding Nrf2 activation and Nrf2-dependent responses in severe cases. This supports the hypothesis that muscle impairment in mild patients is compensated by metabolic adaptations that help to counteract oxidative stress and inflammation. The role of oxidative stress in FSHD pathology is supported by clinical studies, showing elevated oxidative stress markers in FSHD muscle tissue [100], as well as by experimental studies on myoblasts, demonstrating that DUX4-induced oxidative stress contributes to aberrant differentiation [23]. Furthermore, oxidative stress and DNA damage have been identified as upstream inducers of DUX4 expression in FSHD myocytes [101] and can be considered risk factors for disease onset or progression. Notably, NAD(H)/NADP(H) levels and their balance played crucial roles in stress responses and redox homeostasis [102,103,104]. Redox cofactors showed differences between mild and severe patients with NAD(H)/NADP(H) couples decreased in severe cases, while levels of NAD(H) were increased in mild patients and NADP(H) levels were not significantly reduced compared to controls.

Due to the decrease in NNT, severe cases relied solely on cytosolic citrate. The activation of the reductive PPP pathway and TDK and TALDO overexpression [105,106] induced erythritol synthesis. These molecules were at variance between mild and severe cases.

Furthermore, in severe cases decreased levels of enzymes regulating N- and O-protein glycosylation and increased hyaluronic acid synthesis promoted the inhibition of autophagy and survival, as confirmed by increased levels of p53, CASP8 and CASP1 gene transcripts and decreased protein levels of LAMP2 [105,107,108,109]. Many subunits of the 20S and 26S proteasome are increased (see the Supplementary Materials), along with several heat shock proteins, particularly HSP90AB1, which is a marker of ER stress. These changes align with findings from animal models [110] and confirm impaired autophagy and proteostasis [111].

Collectively, these results may indicate that muscle weakness in severe FSHD patients is associated with an inhibition of glycolysis followed by the activation of reductive PPP, lack of HBP, and activation of the reductive TCA cycle, culminating into reductive stress. The latter is exacerbated by a deficiency in the pool of redox cofactors, rendering the increased levels of antioxidant proteins—such as GLRX, GSTP1, PRDX2, PRDX6, and SOD1—ineffective for controlling ROS production associated with HIF1A and NOS3 activation [76,88,94,103,112,113].

Our findings suggest potential therapeutic targets to ameliorate muscle weakness, including the modulation of antioxidant capacity, inhibition of inflammatory pathways, and activation of the HBP by supplementation of a GlcNac precursor to stimulate N- and O-glycosylation, promoting UPR attenuation [87,88]. Additionally, in mild patients, treatment with an antioxidant cocktail [14,68] can be combined with daily exercise, which may help to sustain muscle function, as observed in studies of prolonged bed rest [114] and in FSHD patients [115,116]. In severe cases, our results suggest a first step focused on improving redox cofactors levels and only after replenishing these levels promote treatment with antioxidants capable of entering the mitochondria (e.g., mitoTEMPO and, CoQ10) [68]. This approach may help to alleviate hypoxia signaling and improve metabolic activity in DUX4-expressing myotubes.

Nutritional treatments with antioxidants may enhance the muscle antioxidant capacity; however, careful consideration must be given to patients’ characteristics and the specific molecules used in treatment especially in light of conflicting trial results [68,117,118,119,120]. Targeted investigations in blood and cell cultures are warranted to validate molecules selected in this study for diagnosis and disease monitoring.

In conclusion, our proteomic data indicate that metabolic changes characterize both mild and severe FSHD patients, although to different extents and with different consequences. In mild cases, metabolic reprogramming helps to maintain muscle function by activating compensatory pathways utilizing alternative energy sources. However, in severe cases, these adaptations are absent or insufficient, leading to more profound muscle impairment. Understanding these metabolic differences may provide new insights into potential therapeutic targets for FSHD.

The main limitations of this study lie in the number of samples and small muscle biopsies, limiting confirmatory tests such as the assessment of GSH/GSSG levels in mild and severe cases. Nevertheless, analyzing whole tissue extracts provided a global picture, offering preliminary insights into the intricate crosstalk regulating muscle function and dysfunction in FSHD.

## Data Availability

The identification data for the altered proteins detected by 2D-DIGE and LC–ESI–MS/MS are deposited in the UNIMI dataverse repository and freely downloadable at https://doi.org/10.13130/RD_UNIMI/KTS29V.

## Supplementary Materials

The following supporting information can be downloaded at https://www.mdpi.com/article/10.3390/antiox13111406/s1, Figure S1: Representative histochemistry images of muscle biopsies from a control, a mild FSHD patient, and a severe FSHD patient. Figure S2: Representative 2D-DIGE maps. Figures S3 and S4: Differentially expressed proteins involved in cytoskeletal assembly, microtubule organization, actin modulation, intermediate filaments organization, Z-disk and M-line structures, and thin and thick filaments are shown for mild and severe patients, respectively. Figure S5: Immunoblotting full-length images. Table S1: Patients’ clinical data. Table S2: Commercially available probes for quantitative PCR analysis. Table S3: List of proteins involved in each canonical pathway identified as significantly altered by IPA analysis.

## Abbreviations

2D-DIGE	two-dimensional difference in gel electrophoresis
CTR	control subjects
DTT	dithiothreitol
DUX4	double homeobox 4
FASP	filter-aided sample preparation
FAT1	FAT atypical cadherin 1
FDR	False discovery rate
FRG1	FSHD region gene 1
FSHD	facio scapulo humeral dystrophy
GFAT1	Glutamine-fructose-6-phosphate aminotransferase 1
GLUL	glutamine synthetase
HAS1	hyaluronan synthase 1
HBP	hexosamine biosynthetic pathway
IPA	Ingenuity Pathway Analysis
LAMP2	lysosome-associated membrane protein 2
LC-ESI-MS/MS	liquid chromatography with tandem mass spectrometry
MALDI-ToF	desorption/ionization–time-of-flight
OGA	O-GlcNAcase
OGT	O-GlcNAc transferase
PAX7	Paired Box 7
PMSF	phenylmethanesulfonyl fluoride
PPP	pentose phosphate pathway
SLC25A4	solute carrier family 25 member 4 (ANT1)
ACAA2	3-ketoacyl-CoA thiolase
ACADM	medium-chain specific acyl-CoA dehydrogenase
ACADVL	very long-chain specific acyl-CoA dehydrogenase
ACAT1	acetyl-CoA acetyltransferase
ACO1	cytosolic aconitate hydratase
ACO2	aconitate hydratase
ACSL1	long-chain-fatty-acid-CoA ligase 1
ACTA1	alpha-actin
ACTB	cytoplasmic actin
ACTC1	alpha cardiac muscle actin
ACTN1, ACTN2, ACTN4	alpha-actinin-1, 2 and 4
ADSS1	adenyl succinate synthase
AKR1B1	aldo-keto reductase 1B
ALDOA, ALDOC	fructose-bisphosphate aldolase A and C
AMPD1	AMP deaminase 1
APOA2, APOA4, APOB, APOH	apolipoprotein A-II, A-IV, B-100, H
APRT	adenine phosphoribosyl transferase
ATIC	bifunctional purine biosynthesis enzyme
ATP5F1A, ATP5F1B, ATP5MG	ATP synthase F1 subunit alpha, beta and membrane subunit g
BAG3	BAG family molecular chaperone regulator 3
C1QBP	complement component 1 Q subcomponent-binding protein, mitochondrial
C3, C4B	complement C3 and C4-B
C4BPA	C4b-binding protein alpha chain
CALM1	calmodulin
CASP8, CASP1	caspase 8 and 1
CAT	catalase
CD59	CD59 glycoprotein
CFB, CFH	complement factor B and H
CFL1	cofilin-1
CLU	clusterin
COL1A1, COL3A1, COL4A1, COL5A1, COL14A1, COL15A1, COL18A1	collagen alpha-1 (I) chain, (III) chain, (IV) chain, (V) chain, (XIV) chain, (XV) chain and (XVIII) chain
COL1A2, COL4A2	collagen alpha-2 (I) chain and (IV) chain
COL6A1, COL6A2, COL6A3	collagen alpha-1, alpha-2, and alpha-3 (VI) chain
COQ8A	atypical kinase COQ8A
COX5B, COX6B1, COX7A1	cytochrome c oxidase subunit 5B, 6B1, 7A1
CPT1B	carnitine O-palmitoyltransferase 1
CS	citrate synthase
CSTB	cystatin-B
CYB5R1, CYB5R3	NADH-cytochrome b5 reductase 1 and 3
CYCS	cytochrome C
CYP27A1	sterol 26-hydroxylase
DBI	acyl CoA-binding protein
DCN	decorin
DDT	D-dopachrome decarboxylase
DECR1	2,4-dienoyl CoA reductase
DLAT	dihydrolipoyllysine-residue acetyltransferase
DLD	dihydrolipoyl dehydrogenase
DPT	dermatopontin
ECH1	delta(3,5)-Delta(2,4)-dienoyl-CoA isomerase
ECHS1	enoyl-CoA hydratase
ECI1	enoyl CoA delta isomerase 1
ECM	extracellular matrix
ENO1	alpha-enolase
ENO3	enolase 3
F2	prothrombin
FABP4, FABP5	fatty acid-binding protein
FAK	focal adhesion kinases
FBLN1	fibulin-1
FBN1	fibrillin-1
FBP2	fructose-bisphosphatase 2
FGA, FGB, FGG	fibrinogen a, beta and g chain
FH	fumarate hydratase
FHL1	four-and-a- half LIM domains 1
FLNA	filamin A
FLNC	filamin-C
FN1	fibronectin
GAPDH	glyceraldehyde-3-phosphate dehydrogenase
GATD3	glutamine amidotransferase-like class 1 domain-containing protein 3
GLRX	cytosolic glutaredoxin-1
GLS	gelsolin
GLUD1	mitochondrial glutamate dehydrogenase 1
GOT1, GOT2	cytoplasmic and mitochondrial aspartate aminotransferase
GPI	glucose-6-phosphate isomerase
GSTM2	cytosolic glutathione S-transferase mu 2
GSTP1	cytosolic glutathione S-transferase P
H6PD	hexose-6-phosphate dehydrogenase
HADH	hydroxyacyl-coenzyme A dehydrogenase
HADHA	trifunctional enzyme subunit alpha
HAGH	mitochondrial hydroxyacylglutathione hydrolase
HEBP2	heme-binding protein 2
HIF1A, HIF3A	hypoxia inducible factor 1 and 3 subunit alpha
HIF1AN	HIF1A inhibitor
HP	haptoglobin
HPX	hemopexin
HSD17B10	3-hydroxyacyl-CoA dehydrogenase type-2
HSD17B12	very-long-chain 3-oxoacyl-CoA reductase
HSPA4	heat shock 70 kDa protein 4
HSPB1, HSPB2, HSPB6, HSPB7	heat shock proteins
HSPE1	10 kDa heat shock protein
HSPG2	heparan sulfate proteoglycan 2
ICAM1	intercellular adhesion molecule 1
IDH1	cytoplasmic NADP+-dependent isocitrate dehydrogenase
IDH2	mitochondrial NADP+-dependent isocitrate dehydrogenase
IDH3A	mitochondrial isocitrate dehydrogenase [NAD] subunit alpha
IGHA1	Ig alpha-1 chain C region
IGHG1, IGHG2, IGHG3, IGHG4	Ig gamma C region 1, 2, 3, 4 chain
IGHM	Ig mu chain C region
IGKC	Ig kappa chain C region
IGLC6	Ig lambda-6 chain C region
IL1B, IL1R1	interleukin 1 beta and receptor type 1
IL6R	interleukin 6 receptor
KLHL41	Kelch-like protein 41
KNG1	kininogen-1
LAMA2	laminin 2
LAMA4, LAMA5, LAMB1, LAMB2, LAMC1	laminin subunit alpha-4, alpha-5, beta-1, beta-2, gamma-1
LDHA, LDHB	L-lactate dehydrogenase A chain and B chain
LGALS1	galectin-1
LMNA	pre-laminin
LUM	lumican
MDH1	cytosolic malate dehydrogenase
MDH2	malate dehydrogenase 2
MHC	major histocompatibility complex
MIF	macrophage migration inhibitory factor
MYBPC1, MYBPC2	myosin-binding protein C slow and fast type
MYH11	myosin-11
MYH2	myosin-2
MYH9	myosin-9
MYL6	myosin light polypeptide 6
MYL9	myosin regulatory light polypeptide 9
MYOM1, MYOM2, MYOM3	myomesin subunits
MYOT	myotilin
NDUFA5, NDUFA6	NADH dehydrogenase [ubiquinone] 1 alpha subcomplex subunit 5 and 6
NDUFS3, NDUFS4, NDUFS5, NDUFS6, NDUFS8	NADH dehydrogenase [ubiquinone] iron-sulfur protein 3, 4, 5, 6, 8
NDUFV2	NADH dehydrogenase [ubiquinone] flavoprotein 2
NEB	nebulin
NID1, NID2	nidogen-1 and -2
NNT	NAD(P) transhydrogenase mitochondrial
NOS3	nitric oxide synthase 3
OGDH	2-oxoglutarate dehydrogenase
PADI2	peptidyl arginine deiminase 2
PARK7	protein deglycase DJ-1
PCMT1	protein-L-isoaspartate (D-aspartate) O-methyltransferase
PDHA1, PDHB	pyruvate dehydrogenase E1 component subunit alpha and beta
PFKM	ATP-dependent 6-phosphofructokinase muscle type
PGAM1, PGAM2	phosphoglycerate mutase 1 and 2
PGD	phosphogluconate dehydrogenase
PGK1	phosphoglycerate kinase 1
PKM	pyruvate kinase
PLIN4	perilipin-4
PNP	purine nucleoside phosphorylase
PPIA	peptidyl-prolyl cis-trans isomerase A
PRDX2	peroxiredoxin 2
PRDX3	thioredoxin-dependent peroxide reductase, mitochondrial
PRDX6	peroxiredoxin 6
PRELP	prolargin
RAP1B	Ras-related protein Rap-1b
S100A10, S100A11, S100A13, S100A4, S100A6, S100A8	protein S100
SDHA	succinate dehydrogenase [ubiquinone] flavoprotein subunit
SDHB	succinate dehydrogenase [ubiquinone] iron-sulfur subunit
SLC25A11	mitochondrial 2-oxoglutarate/malate carrier protein
SLC25A12	calcium-binding mitochondrial carrier protein Aralar1
SLC25A4	ADP/ATP translocase 1
SLC2A1	solute carrier family 2 member 1
SOD1	superoxide dismutase [Cu-Zn]
SOD2	superoxide dismutase [Mn], mitochondrial
ST13	hsc70-interacting protein
SUCLA2	succinyl-CoA ligase [ADP-forming] subunit beta
SYPL2	synaptophysin-like protein 2
TALDO	transaldolase
THBS4	thrombospondin-4
TIMP1	metallopeptidase inhibitor 1
TKT	transketolase
TLN1, TLN2	talin 1 and 2
TMOD1	tropomodulin-1
TNXB	tenascin-X
TPI1	triosephosphate isomerase
TPM4	tropomyosin 4
TRIM72	tripartite motif protein
TTN	titin
TXN	thioredoxin
UQCRC1, UQCRH, UQCRB, UQCR11	cytochrome b-c1 complex subunit 1, 6, 7, 10
VAMP2	vesicle associated membrane protein 2
VCAM1	vascular cell adhesion molecule 1
VCL	vinculin
VEGFA	vascular endothelial growth factor A
VIM	vimentin
WDR1	WD repeat-containing protein 1
