# Supplementary material for: Muscle Proteome Analysis of Facioscapulohumeral Dystrophy Patients Reveals a Metabolic Rewiring Promoting Oxidative/Reductive Stress Contributing to the Loss of Muscle Function
Source: Antioxidants (Basel). 2024 Nov 16;13(11):1406. doi: 10.3390/antiox13111406 (PMC11591206; doi:10.3390/antiox13111406)
Supplement: Supplementary file 1 [file antioxidants-13-01406-s001.zip › Supp Fig 1.pdf]

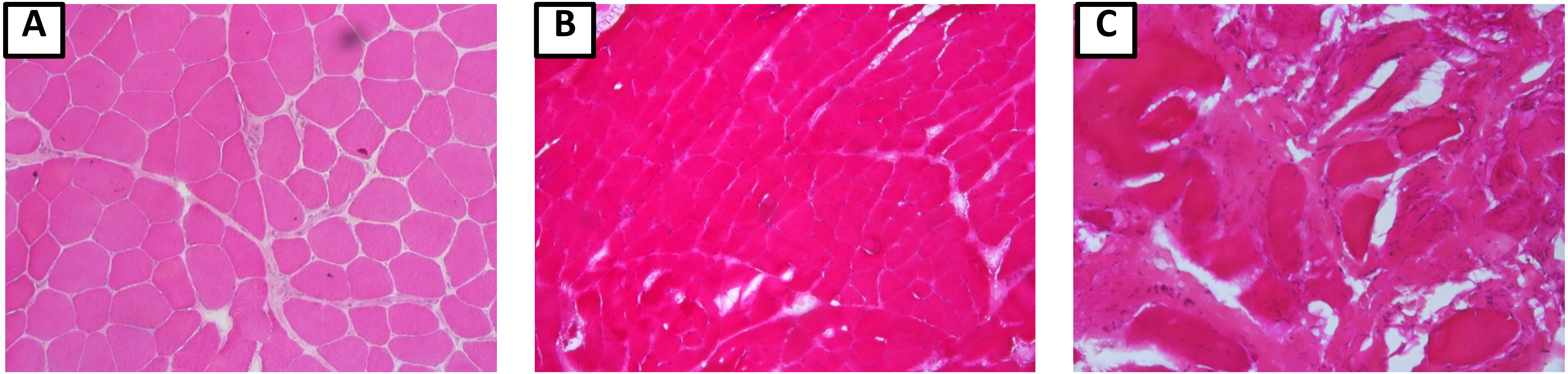

Supplementary Figure 1 shows hematoxylin and eosin-stained sections. **A** Shows the staining of a control sample. **B** Depicts a patient with mild FSHD, where scattered myopathic changes, such as variability in muscle fiber size, fiber splitting, and internalized nuclei, can be observed. **C** Illustrates a patient with severe FSHD, showing widespread degenerative alterations, including muscle fiber atrophy, fibrosis, and fatty infiltration, which are typical of advanced-stage muscular dystrophies.
