# Supplementary material for: Muscle Proteome Analysis of Facioscapulohumeral Dystrophy Patients Reveals a Metabolic Rewiring Promoting Oxidative/Reductive Stress Contributing to the Loss of Muscle Function
Source: Antioxidants (Basel). 2024 Nov 16;13(11):1406. doi: 10.3390/antiox13111406 (PMC11591206; doi:10.3390/antiox13111406)
Supplement: Supplementary file 1 [file antioxidants-13-01406-s001.zip › Supp Fig 2.pdf]

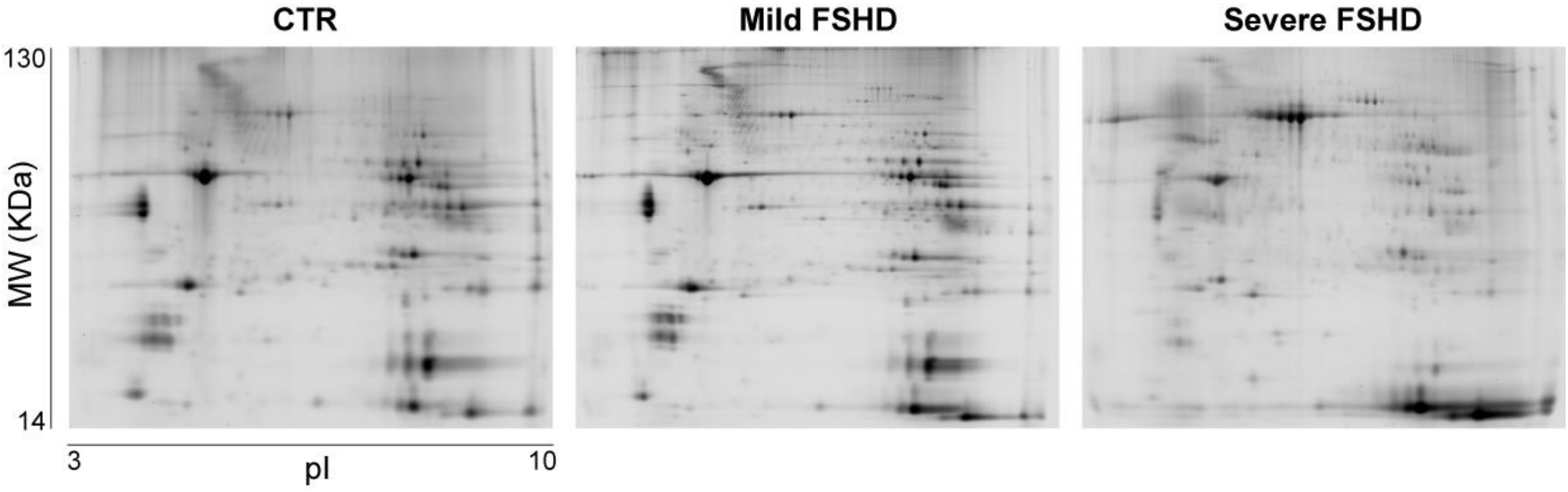

Supplementary Figure 2: Representative 2-D DIGE gel images of Control (CTR), Mild and Severe FSHD. Samples were separated on 24 cm, 3–10 nonlinear pH gradient IPG strips, with a voltage gradient ranging from 200 to 8000 V, for a total of 70 000 VhT. The second dimension was carried out in 20 x 25 cm2, 12% T, 2.5% C constant concentration polyacrylamide gels at 20°C and 15 mA per gel using the Ettan Dalt II system (GE Healthcare).
