# Supplementary material for: Muscle Proteome Analysis of Facioscapulohumeral Dystrophy Patients Reveals a Metabolic Rewiring Promoting Oxidative/Reductive Stress Contributing to the Loss of Muscle Function
Source: Antioxidants (Basel). 2024 Nov 16;13(11):1406. doi: 10.3390/antiox13111406 (PMC11591206; doi:10.3390/antiox13111406)
Supplement: Supplementary file 1 [file antioxidants-13-01406-s001.zip › Supp Fig 3 and 4.pdf]

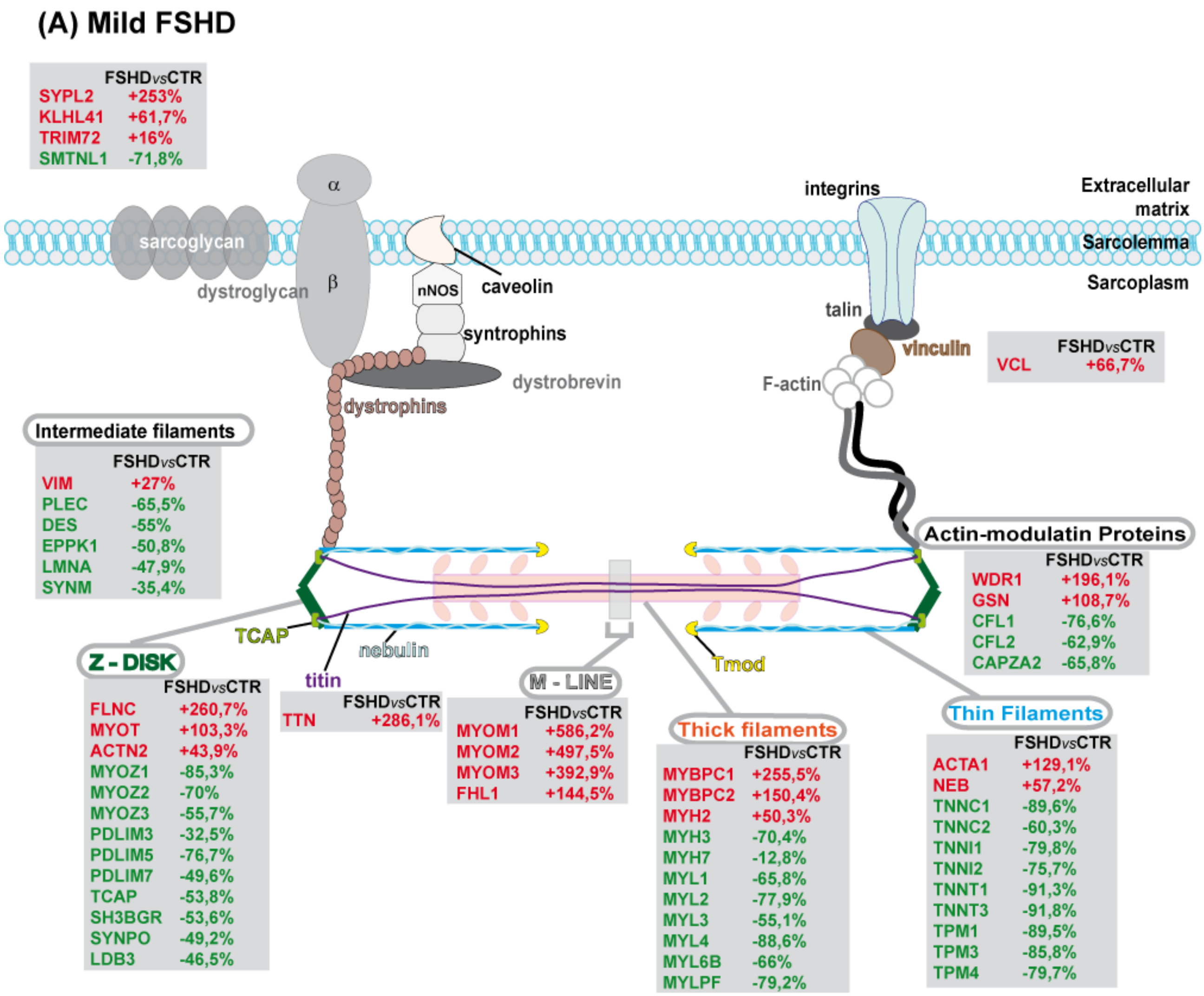

Supplementary figure 3. In mild FSHD patients, the cytoskeletal region synaptophysin-like protein 2 (SYPL2), Kelch-like protein 41(KLHL41), and tripartite motif protein (TRIM72) were increased whereas, smoothelin-like protein 2 (SMTLN1) decreased.

Concerning protein changes related to sarcomeric, muscle regeneration and intermediate filaments, the connection between ECM and myofibers mediated by focal adhesion kinases (FAK) was altered with a vinculin (VCL) increase. Several proteins involved in actin modulation were also dysregulated, WD repeat-containing protein 1 (WDR1) and gelsolin (GSN) increased whereas cofilin-1 and 2 (CFL1, CFL2) and capping actin protein of muscle Z-line subunit alpha 2 (CAPZA2) decreased. Intermediate filaments are characterized by increased levels of vimentin (VIM) and decreased levels of plectin (PLEC), desmin (DES), epiplakin (EPPK1), pre-laminin (LMNA), and synemin (SYNM).

Giant proteins and Z-disk organization were severely dysregulated with a common increased trend for titin (TTN), filamin C (FLNC), myotilin (MYOT), and actinin (ACTN2); whereas, myozenin subunits (MYOZ1, MYOZ2, MYOZ3), four and a half LIM domain protein subunits (PDLIM3, PDLIM5, PDLIM7), titin-cap (TCAP), SH3 domain-binding glutamic acid -rich-like protein (SH3BGRL), synaptopodin (SYNPO), and LIM domain binding 3 (LDB3) decreased.

Concerning proteins localized at the M-Line a significant increase of, myomesin subunits (MYOM1, MYOM2, MYOM3), and four and a half lim domains 1 (FHL1) was detected.

Thick filaments were characterized by increased levels of myosin-binding protein C slow and fast type (MYBPC1, MYBPC2) and of myosin-2 (MYH2); whereas, embrionic myosin heavy chain 3 (MYH3), myosin-7 (MYH7), myosin light chain skeletal muscle isoforms (MYL1, MYL3,

embrionic MYL4, MYL6B, myosin regulatory light chain 2, cardiac and skeletal muscle isoform (MYL2, MYLPF) decreased.

Thin filaments showed increased levels of actin 1 (ACTA1) and nebulin (NEB); while, a decrease of troponin C, slow and fast type (TNNC1, TNNC2), troponin I, slow and fast type (TNNI1, TNNI2), troponin T, slow and fast type (TNNT1, TNNT3), tropomyosin 1, 3 and alpha-4 chain (TPM1, TPM3, TPM4) was detected.

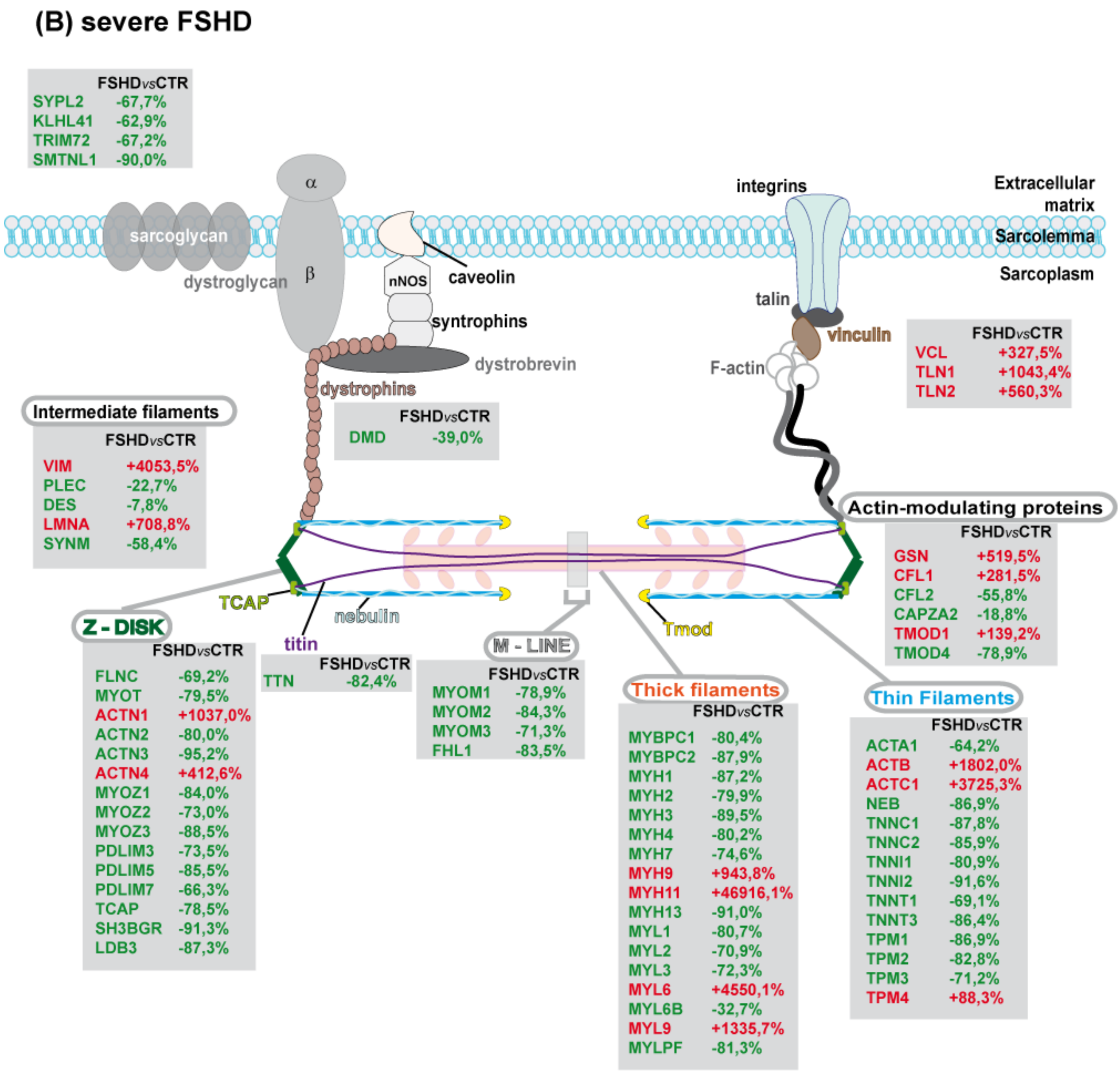

Supplementary figure 4. In severe FSHD, SYPL2, KLHL41, TRIM72 and SMTLN1 decreased. The connection between ECM and myofibers was altered with increased VCL, talin-1 and -2 (TLN1, TLN2). The dystrophin-glycoprotein complex protein dystrophin (DMD) was decreased. Proteins involved in actin modulation were also dysregulated, GSN increased together with CFL1 and tropomodulin-1 (TMOD1), whereas CFL2, CAPZA2 and tropomodulin-4 (TMOD4) decreased. Intermediate filaments were characterized by increased levels of VIM and LMNA and decreased PLEC, DES, and SYNM. Giant proteins and Z-disk organization proteins alpha-actinin 1 and 4 (ACTN1, ACTN4) increased, whereas TTN, FLNC, MYOT, ACTN2, alpha-actinin-3 (ACTN3), MYOZ1, MYOZ2, MYOZ3, PDLIM3, PDLIM5, PDLIM7, TCAP, SH3BGRL, and LDB3 decreased. M-Line proteins MYOM1, MYOM2, MYOM3, and FHL1 were all decreased. Thick filaments were characterized by increased levels of myosin-9 (MYH9), myosin-11 (MYH11), myosin light polypeptide 6 (MYL6), and myosin regulatory light polypeptide 9 (MYL9), whereas MYBPC1, MYBPC2, myosin-1 (MYH1), MYH2, MYH3, myosin-4 (MYH4), MYH7, myosin-13 (MYH13), MYL1, MYL2, MYL3, MYL6B, and MYLPF decreased. Thin filaments showed increased levels of actin, cytoplasmic 1 (ACTB), actin, alpha cardiac muscle 1 (ACTC1) and tropomyosin-4 (TPM4), while a decrease of ACTA1, NEB, TNNC1, TNNC2, TNNI1, TNNI2, TNNT1, TNNT3, TPM1, tropomyosin beta chain (TPM2) and TPM3 was detected.
