# Supplementary material for: Muscle Proteome Analysis of Facioscapulohumeral Dystrophy Patients Reveals a Metabolic Rewiring Promoting Oxidative/Reductive Stress Contributing to the Loss of Muscle Function
Source: Antioxidants (Basel). 2024 Nov 16;13(11):1406. doi: 10.3390/antiox13111406 (PMC11591206; doi:10.3390/antiox13111406)
Supplement: Supplementary file 1 [file antioxidants-13-01406-s001.zip › Supp Fig 5.pptx]

## Slide 1
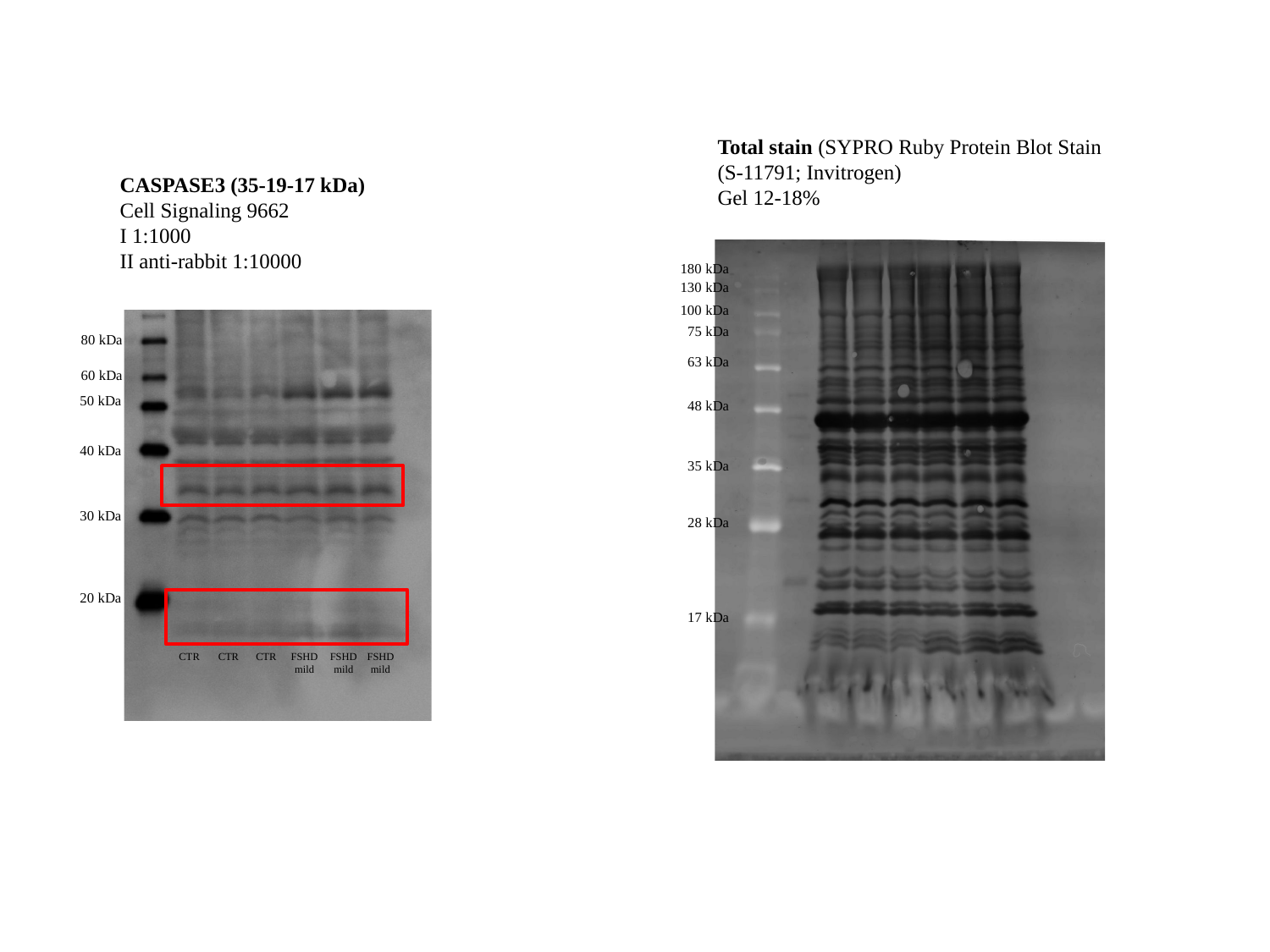

Total stain (SYPRO Ruby Protein Blot Stain (S-11791; Invitrogen)
Gel 12-18%
CASPASE3 (35-19-17 kDa)
Cell Signaling 9662
I 1:1000
II anti-rabbit 1:10000
180 kDa
130 kDa
100 kDa
75 kDa
63 kDa
48 kDa
35 kDa
28 kDa
17 kDa
80 kDa
60 kDa
50 kDa
40 kDa
30 kDa
20 kDa
CTR
CTR
CTR
FSHD
mild
FSHD
mild
FSHD
mild

## Slide 2
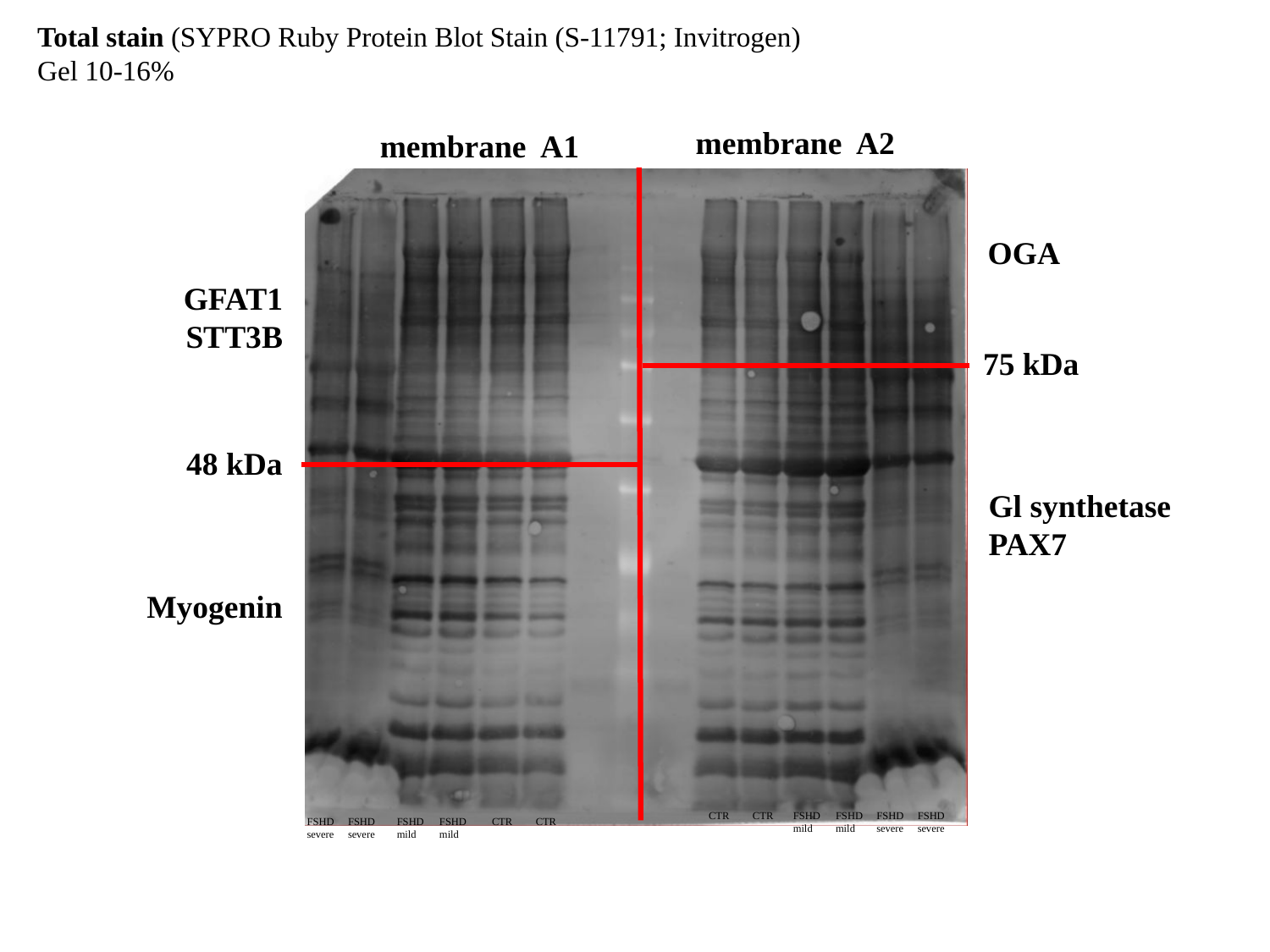

Total stain (SYPRO Ruby Protein Blot Stain (S-11791; Invitrogen)
Gel 10-16%
membrane A2
membrane A1
OGA
GFAT1
STT3B
75 kDa
48 kDa
Myogenin
CTR
CTR
FSHD
mild
FSHD
mild
FSHD
severe
FSHD
severe
FSHD
severe
FSHD
severe
FSHD
mild
FSHD
mild
CTR
CTR
Gl synthetase
PAX7

## Slide 3
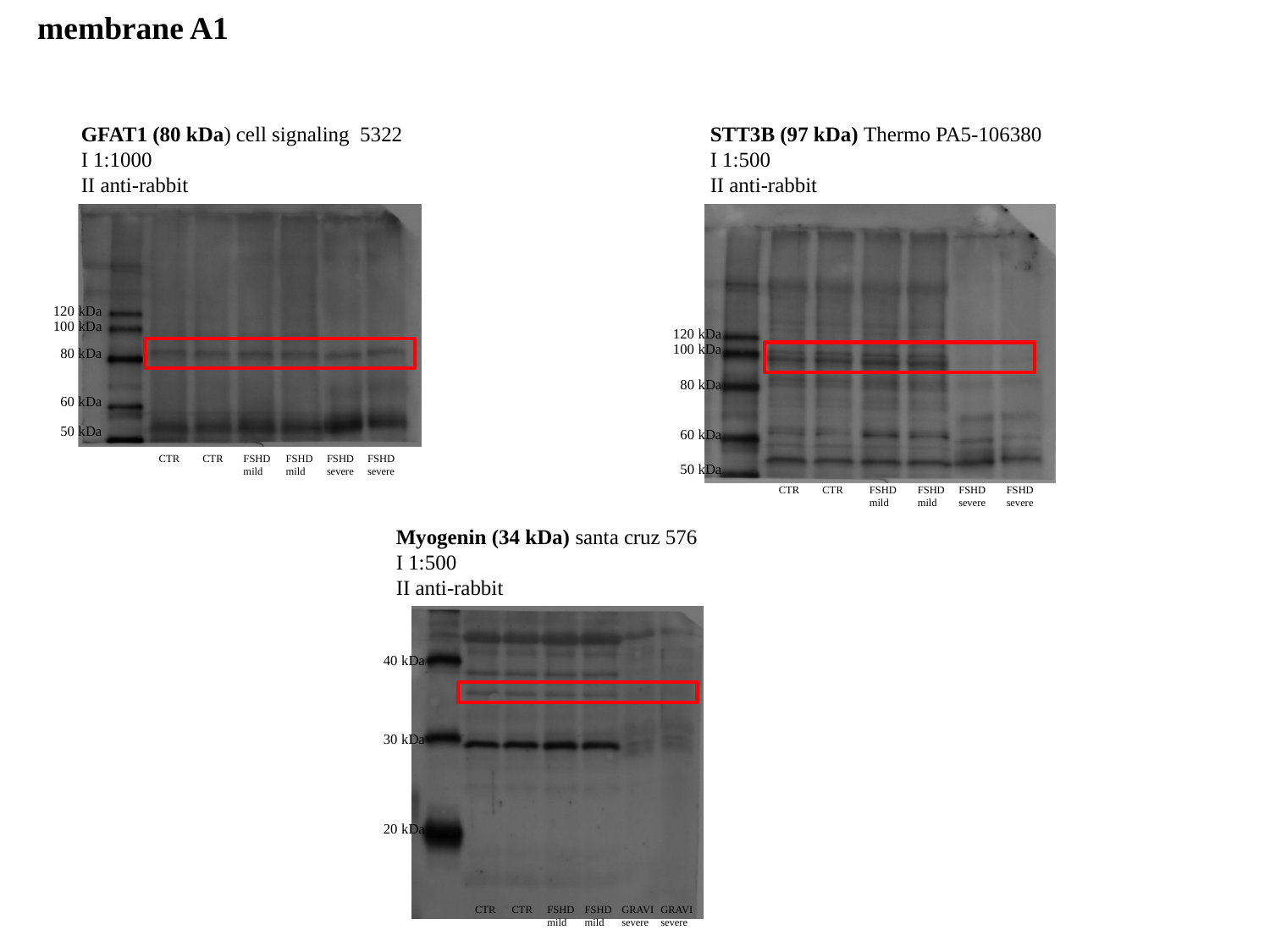

membrane A1
GFAT1 (80 kDa) cell signaling 5322
I 1:1000
II anti-rabbit
STT3B (97 kDa) Thermo PA5-106380
I 1:500
II anti-rabbit
120 kDa
100 kDa
80 kDa
60 kDa
50 kDa
CTR
CTR
FSHD
mild
FSHD
mild
FSHD
severe
FSHD
severe
120 kDa
100 kDa
80 kDa
60 kDa
50 kDa
CTR
CTR
FSHD
mild
FSHD
mild
FSHD
severe
FSHD
severe
Myogenin (34 kDa) santa cruz 576
I 1:500
II anti-rabbit
40 kDa
30 kDa
20 kDa
CTR
CTR
FSHD
mild
FSHD
mild
GRAVI
severe
GRAVI
severe

## Slide 4
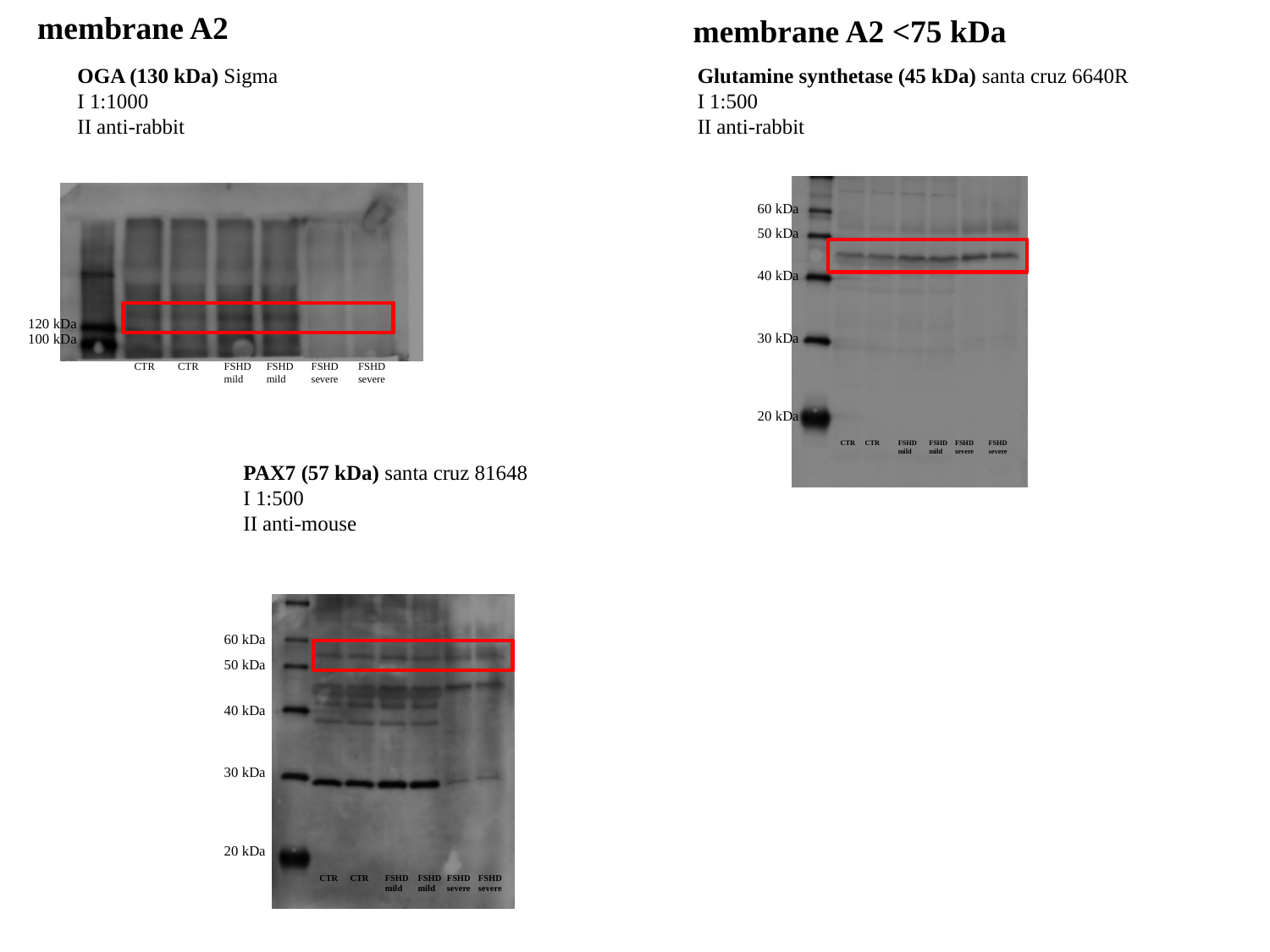

membrane A2
membrane A2 <75 kDa
OGA (130 kDa) Sigma
I 1:1000
II anti-rabbit
Glutamine synthetase (45 kDa) santa cruz 6640R
I 1:500
II anti-rabbit
60 kDa
50 kDa
40 kDa
30 kDa
20 kDa
CTR
CTR
FSHD
mild
FSHD
mild
FSHD
severe
FSHD
severe
120 kDa
100 kDa
CTR
CTR
FSHD
mild
FSHD
mild
FSHD
severe
FSHD
severe
PAX7 (57 kDa) santa cruz 81648
I 1:500
II anti-mouse
60 kDa
50 kDa
40 kDa
30 kDa
20 kDa
CTR
CTR
FSHD
mild
FSHD
mild
FSHD
severe
FSHD
severe

## Slide 5
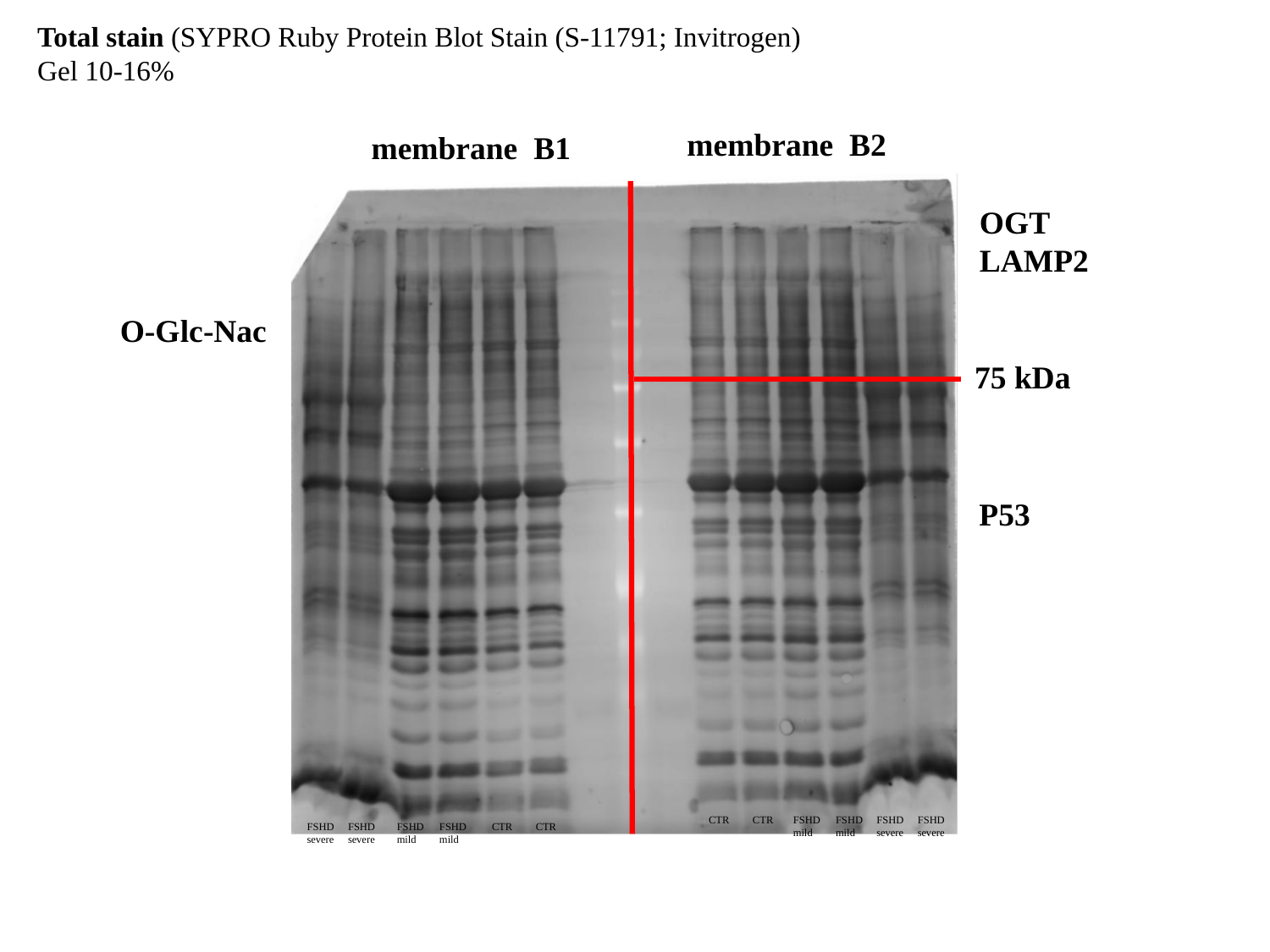

Total stain (SYPRO Ruby Protein Blot Stain (S-11791; Invitrogen)
Gel 10-16%
membrane B2
membrane B1
OGT
LAMP2
O-Glc-Nac
75 kDa
P53
CTR
CTR
FSHD
mild
FSHD
mild
FSHD
severe
FSHD
severe
FSHD
severe
FSHD
severe
FSHD
mild
FSHD
mild
CTR
CTR

## Slide 6
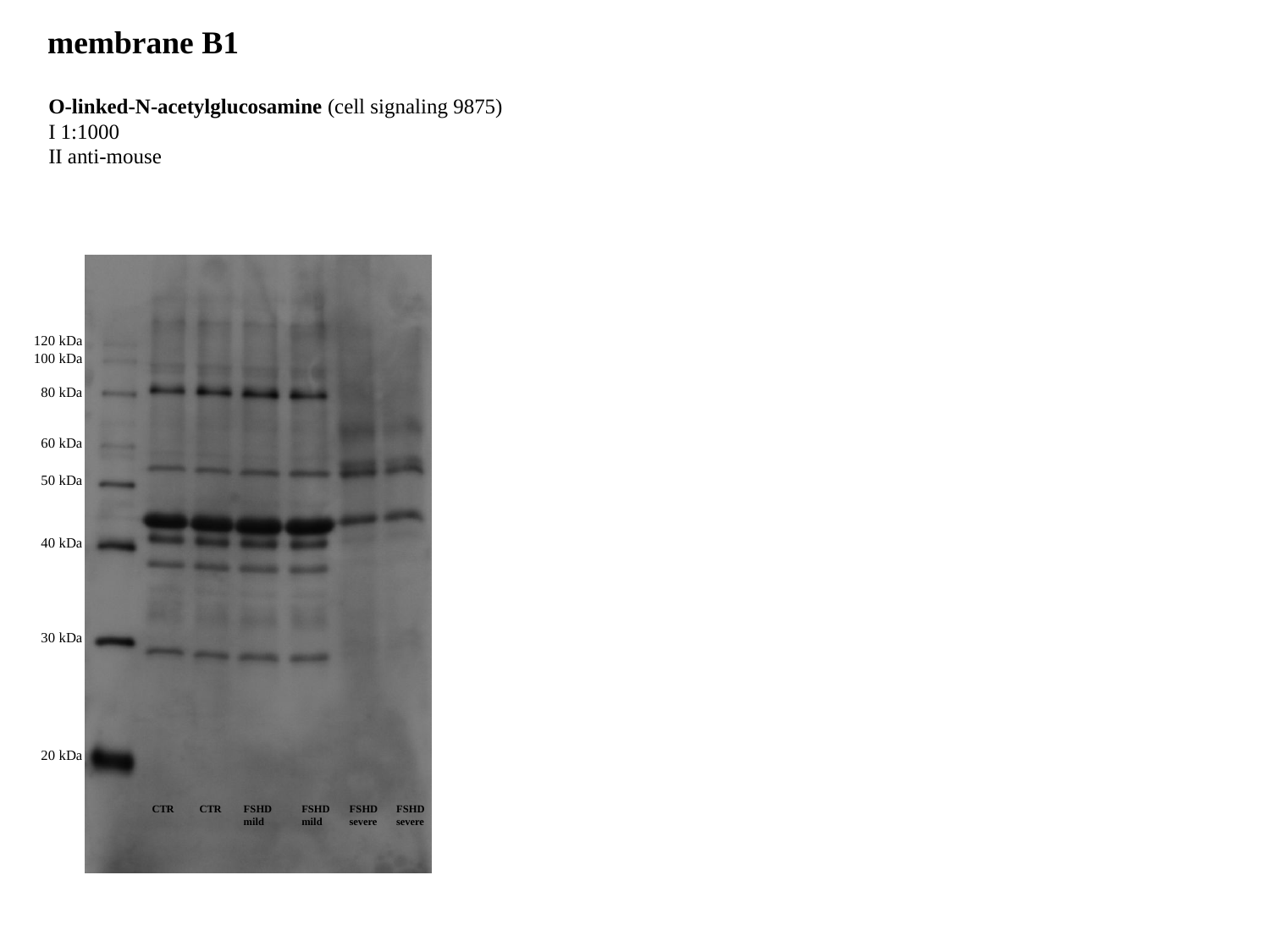

membrane B1
O-linked-N-acetylglucosamine (cell signaling 9875)
I 1:1000
II anti-mouse
120 kDa
100 kDa
80 kDa
60 kDa
50 kDa
40 kDa
30 kDa
20 kDa
CTR
CTR
FSHD
mild
FSHD
mild
FSHD
severe
FSHD
severe

## Slide 7
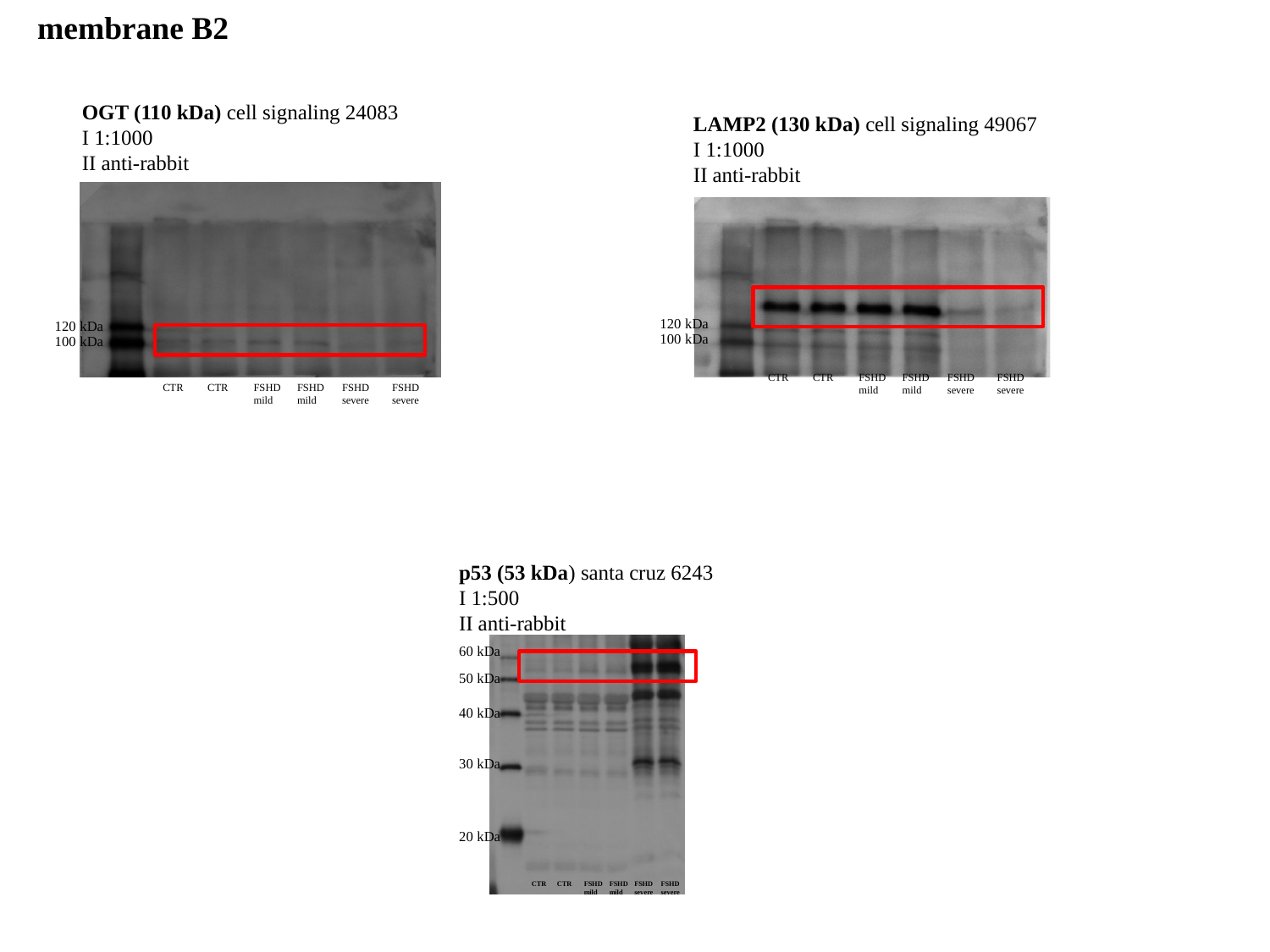

membrane B2
OGT (110 kDa) cell signaling 24083
I 1:1000
II anti-rabbit
LAMP2 (130 kDa) cell signaling 49067
I 1:1000
II anti-rabbit
120 kDa
100 kDa
CTR
CTR
FSHD
mild
FSHD
mild
FSHD
severe
FSHD
severe
120 kDa
100 kDa
CTR
CTR
FSHD
mild
FSHD
mild
FSHD
severe
FSHD
severe
p53 (53 kDa) santa cruz 6243
I 1:500
II anti-rabbit
60 kDa
50 kDa
40 kDa
30 kDa
20 kDa
CTR
CTR
FSHD
mild
FSHD
mild
FSHD
severe
FSHD
severe

## Slide 8
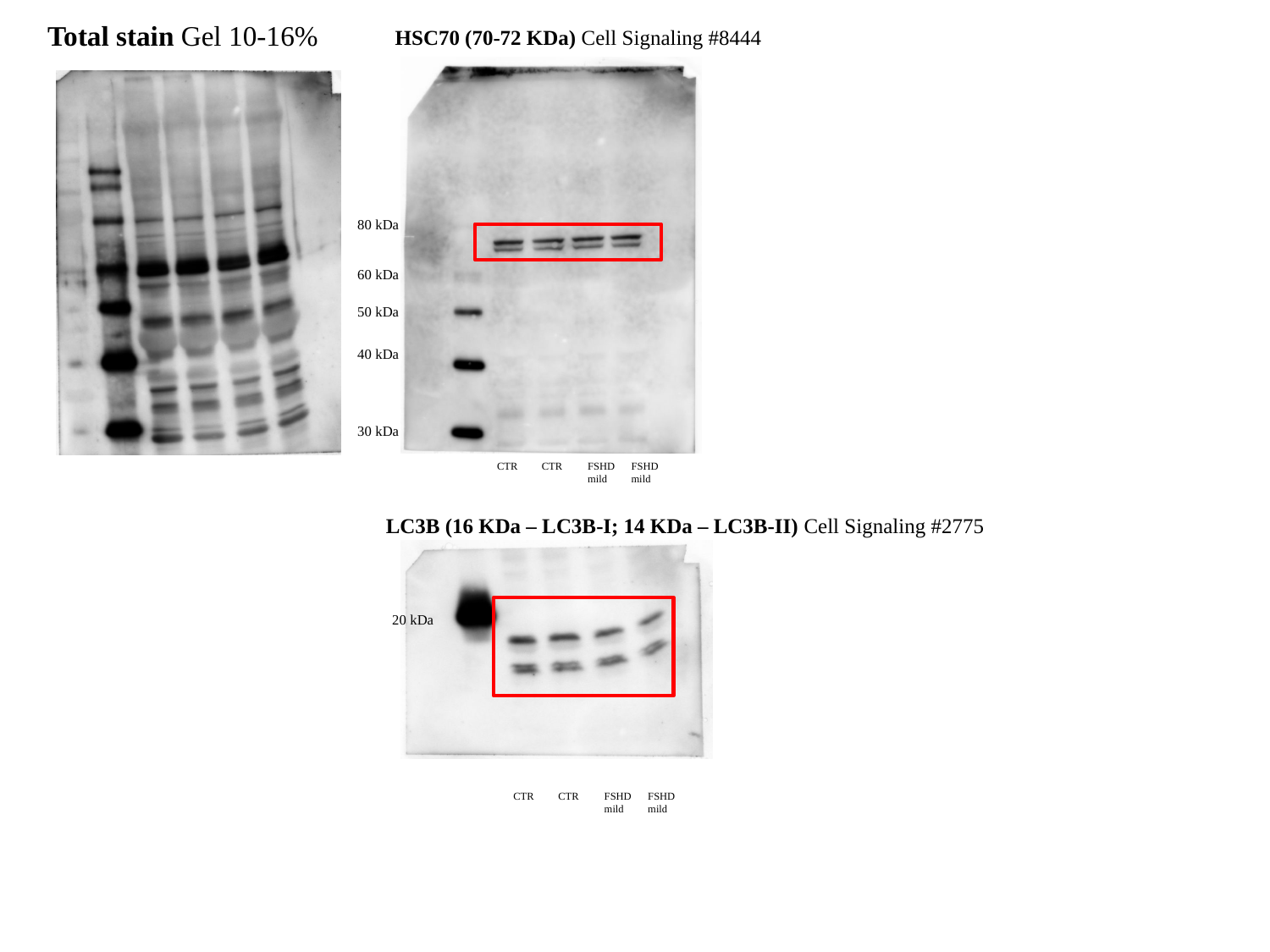

Total stain Gel 10-16%
HSC70 (70-72 KDa) Cell Signaling #8444
80 kDa
60 kDa
50 kDa
40 kDa
30 kDa
CTR
CTR
FSHD
mild
FSHD
mild
LC3B (16 KDa – LC3B-I; 14 KDa – LC3B-II) Cell Signaling #2775
20 kDa
CTR
CTR
FSHD
mild
FSHD
mild

## Slide 9
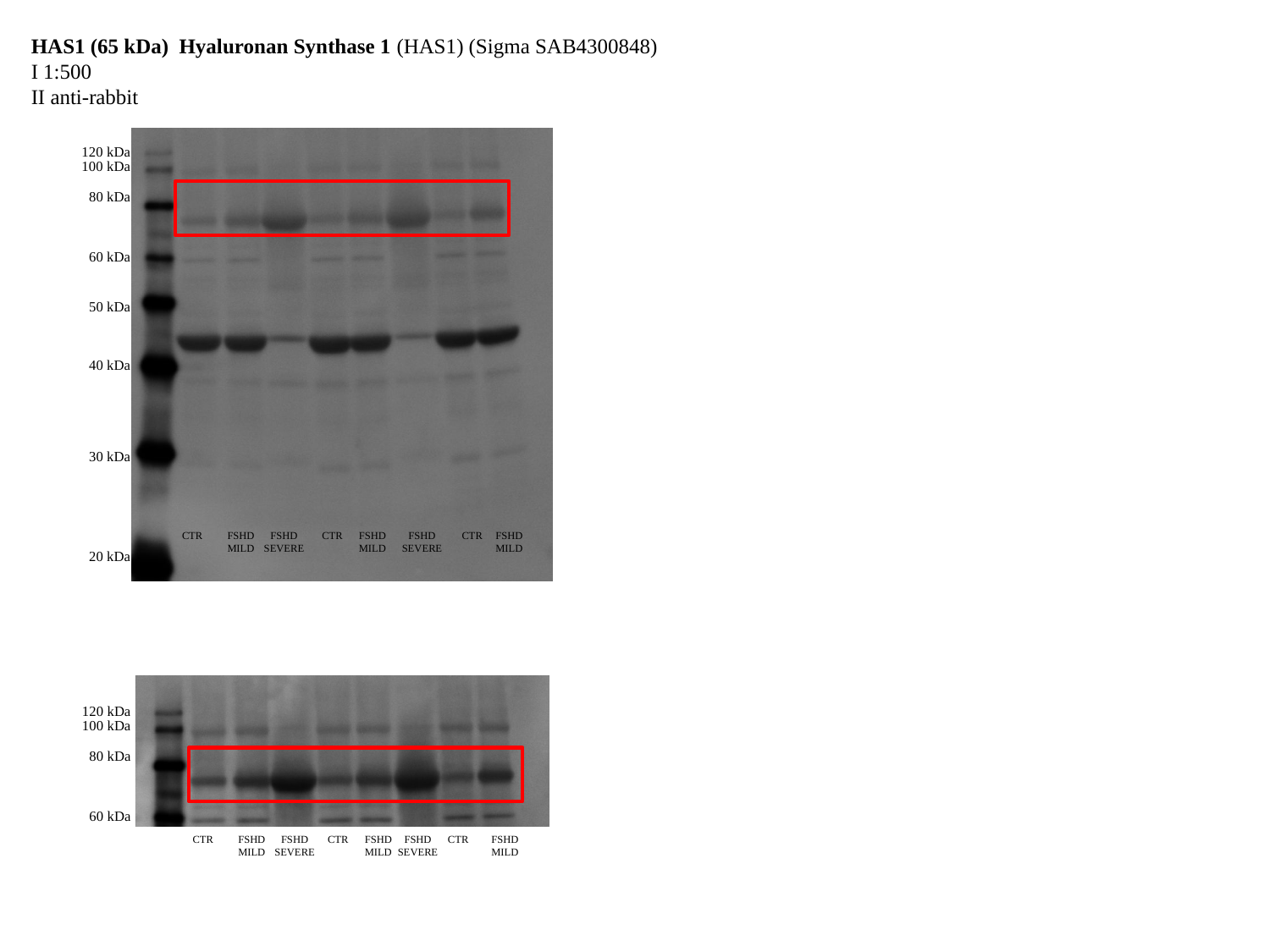

HAS1 (65 kDa) Hyaluronan Synthase 1 (HAS1) (Sigma SAB4300848)
I 1:500
II anti-rabbit
120 kDa
100 kDa
80 kDa
60 kDa
50 kDa
40 kDa
30 kDa
CTR
FSHD
MILD
FSHD
SEVERE
CTR
FSHD
MILD
FSHD
SEVERE
CTR
FSHD
MILD
20 kDa
120 kDa
100 kDa
80 kDa
60 kDa
CTR
FSHD
MILD
FSHD
SEVERE
CTR
FSHD
MILD
FSHD
SEVERE
CTR
FSHD
MILD
